# Phosphorylation of BK channels modulates the sensitivity to hydrogen sulfide (H_2_S)

**DOI:** 10.3389/fphys.2014.00431

**Published:** 2014-11-12

**Authors:** Guzel F. Sitdikova, Roman Fuchs, Verena Kainz, Thomas M. Weiger, Anton Hermann

**Affiliations:** ^1^Department of Physiology of Man and Animals, Kazan Federal UniversityKazan, Russia; ^2^Neurosignaling Unit, Department of Organismic Biology, University of SalzburgSalzburg, Austria; ^3^Division of Cellular and Molecular Neurobiology, Department of Cell Biology, University of SalzburgSalzburg, Austria

**Keywords:** gasotransmitters, hydrogen sulfide (H_2_S), maxi calcium-activated potassium (BK) channels, patch clamp, phosphorylation, GH cells

## Abstract

**Introduction:** Gases, such as nitric oxide (NO), carbon monoxide (CO), or hydrogen sulfide (H_2_S), termed gasotransmitters, play an increasingly important role in understanding of how electrical signaling of cells is modulated. H_2_S is well-known to act on various ion channels and receptors. In a previous study we reported that H_2_S increased calcium-activated potassium (BK) channel activity.

**Aims:** The goal of the present study is to investigate the modulatory effect of BK channel phosphorylation on the action of H_2_S on the channel as well as to recalculate and determine the H_2_S concentrations in aqueous sodium hydrogen sulfide (NaHS) solutions.

**Methods:** Single channel recordings of GH3, GH4, and GH4 STREX cells were used to analyze channel open probability, amplitude, and open dwell times. H_2_S was measured with an anion selective electrode.

**Results:** The concentration of H_2_S produced from NaHS was recalculated taking pH, temperature salinity of the perfusate, and evaporation of H_2_S into account. The results indicate that from a concentration of 300 μM NaHS, only 11–13%, i.e., 34–41 μM is effective as H_2_S in solution. GH3, GH4, and GH4 STREX cells respond differently to phosphorylation. BK channel open probability (Po) of all cells lines used was increased by H_2_S in ATP-containing solutions. PKA prevented the action of H_2_S on channel Po in GH4 and GH4 STREX, but not in GH3 cells. H_2_S, high significantly increased Po of all PKG pretreated cells. In the presence of PKC, which lowers channel activity, H_2_S increased channel Po of GH4 and GH4 STREX, but not those of GH3 cells. H_2_S increased open dwell times of GH3 cells in the absence of ATP significantly. A significant increase of dwell times with H_2_S was also observed in the presence of okadaic acid.

**Conclusions:** Our results suggest that phosphorylation by PKG primes the channels for H_2_S activation and indicate that channel phosphorylation plays an important role in the response to H_2_S.

## Introduction

Hydrogen sulfide (H_2_S), although it is extremely toxic in higher concentrations, similar to the other established gasotransmitters, nitric oxide (NO) and carbon monoxide (CO), serves as a cellular signaling molecule in low concentrations being involved in a vast variety of physiological actions (Goodwin et al., 1989; Abe and Kimura, 1996; Nagai et al., 2004; Kimura et al., 2010; Olson and Whitfield, 2010; Tang et al., 2013) reviewed in (Mustafa et al., 2009; Gadalla and Snyder, 2010; Wang, 2010, 2012, 2014; Hu et al., 2011; Hermann et al., 2012b; Kimura et al., 2012; Kabil et al., 2014) and pathophysiological incidences (i.e., Kimura and Kimura, 2004; Kimura et al., 2012; Mani et al., 2014). H_2_S is endogenously produced in living cells from the amino acid cysteine through various enzymatic pathways (Łowicka and Bełtowski, 2007; Ishigami et al., 2009; Shibuya et al., 2009, 2013; Kimura, 2011; Renga, 2011). Although H_2_S is well-known to modulate receptors and ion channels, such as NMDA receptors (Abe and Kimura, 1996), ATP (adenosine triphosphate)-dependent potassium (K^+^) channels (Zhao and Wang, 2002; Jiang et al., 2010; Tang et al., 2010; Liang et al., 2011; Liu et al., 2011) or calcium (Ca^2+^) channels (Kawabata et al., 2007; García-Bereguiaín et al., 2008; Sun et al., 2008; Maeda et al., 2009; Skovgaard et al., 2011; Matsunami et al., 2012), it was only recently shown to modulate maxi Ca^2+^-activated K^+^ (BK) channels (Telezhkin et al., 2009, 2010; Sitdikova et al., 2010; Jackson-Weaver et al., 2011; Zhao et al., 2013).

The physiological concentrations of H_2_S concentrations are presently under discussion. Previous *in vivo* concentrations reported in the range of 40–160 μM H_2_S (Goodwin et al., 1989; Savage and Gould, 1990) were possibly overestimated since H_2_S derived from various sources were included in the measurements (discussed in Kimura et al., 2012). Recent studies indicate tissue/plasma or blood H_2_S levels of nanomolar (~15 nM in mouse brain/liver homogenates (Furne et al., 2008) to low micromolar (0.4–0.9 μM in rat blood (Wintner et al., 2010); cerebrospinal fluid, pig, ~600 nM (Leffler et al., 2011); ~32 μM in mouse blood (Peng et al., 2011); 34 μM in mouse plasma, (Li et al., 2005) which could rise to low micromolar quantities during pathophysiological conditions (i.e., during hypercapnia in cerebrospinal fluid, pig, 4–5 μM (Leffler et al., 2011). The actual H_2_S concentrations at the target sites are unknown, however, since exogenous H_2_S is highly volatile, rapidly removed, bound or metabolized (cf. Szabó, 2007; Whitfield et al., 2008; Wintner et al., 2010). These H_2_S levels therefore may be of limited relevance to the effective H_2_S concentrations at the target site(s). Nevertheless, action appeared required to carefully estimate the H_2_S concentrations derived from the H_2_S donor (sodium hydrogen sulfide, NaHS) used in our experiments.

BK channels are present in a great variety of non-excitable and excitable cells. Recent detailed studies of BK channels created a vast amount of knowledge regarding their biophysical, structural and functional, physiological, pathophysiological, and pharmacological properties (reviewed in: Ghatta et al., 2006; Salkoff et al., 2006; Cui et al., 2009; Berkefeld et al., 2010; Cui, 2010; Grimm and Sansom, 2010; Hill et al., 2010; Lee and Cui, 2010; Stojilkovic et al., 2010; Wu et al., 2010; Hermann et al., 2012a). BK channels are essential in controlling electrical activity of cells, hormone secretion, vasoregulation, auditory tuning of the hair cells or circadian rhythm generation and participate in mediating drug actions such as ethanol or acetaldehyde. Mutations of the BK channel protein are involved in disorders such as epilepsy, paroxysmal movements, or in erectile dysfunctions. BK channels are synergistically gated by both Ca^2+^ as well as by membrane voltage and are modulated by a wide variety of intra- and extracellular factors, including protein kinases, phosphatases causing phosphorylation/dephosphorylation or changes in their redox environment in particular at the C-terminal region (Reinhart et al., 1991; Levitan, 1994; Tian et al., 2001; Zhou et al., 2001, 2010; Weiger et al., 2002; Lu et al., 2006; Hou et al., 2009; for reviews see Wang, 2008; Dai et al., 2009; Hermann et al., 2012a). Protein kinase G (PKG) in many tissues acts as BK channel activator (Alioua et al., 1998; Kyle et al., 2013), protein kinase A (PKA) can act both ways—activating as well as inhibitory depending on the splice variant of the channel (Hall and Armstrong, 2000; Tian et al., 2001, 2004; Zhou et al., 2001), while protein kinase C (PKC) in many cases acts inhibitory (Shipston and Armstrong, 1996; Schubert and Nelson, 2001; Tian et al., 2004; Kizub et al., 2010; Zhou et al., 2010, 2012; van Welie and du Lac, 2011). PCK-dependent conditional phosphorylation of the channels may be important for PKA-dependent activation (Widmer et al., 2003) or specific isoforms of PKC (PKCδ) may up-regulate BK channel activity (Barman et al., 2004; Kim and Park, 2008). BK channels are also a target of gasotransmitters such as NO and CO which act via intracellular signaling or directly at the channels (reviewed in Hermann et al., 2012c). However, the interaction of BK channel phosphorylation and hydrogen sulfide has not been studied.

In previous experiments we found that H_2_S caused a concentration dependent and reversible increase of membrane outward currents in whole cell experiments (Sitdikova et al., 2010). In single channel recordings H_2_S reversibly increased BK channel open probability in a voltage-dependent, but Ca^2+^ independent manner. Redox modulation of the channels further indicated that the augmenting effect of H_2_S on BK channel activity may be linked to its reducing action on sulfhydryl groups of the channel protein. The aim of the present study was to evaluate the effects of BK channel phosphorylation in the context of H_2_S application employing patch clamp recordings at different types of rat GH pituitary tumor cells (GH3, GH4, and GH4 STREX). We used these three different but related cell lines, because they all express BK channels who differ in their sequences and respond differently to phosphorylation or in the responsiveness to arachidonic acid. The sequences of BK α-subunits in GH3 and GH4 splice variants differ most prominently by the presence or absence of 27 amino acids in the COOH terminus of the channel (Li et al., 2010). In contrast GH4 STREX cells which express a cysteine rich 59 amino-acid insert in the channel tail, contain an additional PKA phosphorylation site (Xie and McCobb, 1998; Tian et al., 2001).

Since BK channels variants of these cell lines respond differently to phosphorylation it appeared interesting to study the impact of H_2_S on these channels types. From our results we hypothesize that the state of BK channel phosphorylation plays an important role in the response to H_2_S.

## Materials and methods

### Cells

We used GH3, GH4C1, and GH4 STREX, rat pituitary tumor cells, for investigation of BK channels. GH cells secrete growth hormone (somatotropin) and prolactin and express various receptors. Stressors applied to GH cells lead to the expression of a 59 amino acid cysteine rich insert at the pore forming α-subunit C-terminus of BK channels—termed GH STREX cells (Xie and McCobb, 1998; Erxleben et al., 2002). In our case the BK-STREX variant was induced by growing GH4/C1 cells in culture medium supplemented with phenol red (10 mg/ml) for at least 10 days. Once the cell line has been established cells can be kept permanently in this medium (Hall and Armstrong, 2000; Erxleben et al., 2002).

Techniques for cell culturing, electrophysiology, and standard solutions used have been described in detail previously (Sitdikova et al., 2010). In brief: cells were cultured at 37°C and 90% humidity in MEM (Minimal Essential Medium), supplemented with 7% fetal calf serum (FCS) and 3% horse serum (HS) for GH3 cells, and HAM F10 plus L-glutamine supplemented with 2.5% FCS and 15% HS for GH4 cells. For experiments cells were grown on poly-D-lysine coated coverslips and used for recordings 3 to 4 days after seeding. Culture media were from Sigma (Vienna, Austria), and sera from Invitrogen (Lofer, Austria), all other chemicals were from Sigma.

### Electrophysiology

In brief: pipettes for single channel patch clamp recordings were drawn from borosilicate glass (Science Products/FRG) with resistances of 3–6 MegaOhm (cf. Sitdikova et al., 2010). As reference electrode an agar bridge containing a silver/silver chloride (Ag/AgCl) pellet was used to avoid H_2_S reaction with Ag to silver sulfide (Ag_2_S) and hence destabilization of the reference electrode. Recordings from excised outside-out patches were made with an Axopatch-200B amplifier connected to a Digidata 1322A interface using pClamp10 software for data acquisition and analysis. Data were filtered at 1 kHz offline and analyzed with Clampfit software (Axon Instruments/Molecular Devices, USA). Raw recordings were idealized with the built in feature of pClamp's Clampfit module using the half-amplitude threshold method with automatic baseline and level tracking. Dwell time analysis was done by fitting open dwell time distributions to standard exponentials with the appropriate number of terms to get an optimal fit. Since BK channels are localized in clusters in cell membranes, all of our patches contained more than one channel. For this reason we analyzed only open dwell times but not closed channel kinetics. Single channel current amplitudes were calculated by fitting amplitude histograms to a Gaussian distribution. Channel open probability was expressed as P_open_ = NPo/n, where NPo = [(to)/(to + tc)], Po = open probability for one channel, to = sum of open times, tc = sum of closed times, N = actual number of channels in the patch, and n = maximum number of individual channels observed in the patch. All equations used were standard built in equations from Clampfit. Experiments were repeated at least three times and the mean, as well as the s.e.m. (standard error of the mean) were calculated.

Experimental solutions were applied via a gravity-driven, electronically switched perfusion system (ALA Scientific Instruments, USA). For rapid solution exchange (about 300–500 ms) membrane patches were held in a stream of the experimental solution from a second pipette.

### Solutions and chemicals

The standard experimental bath solution contained in mM: 145 NaCl, 5 KCl, 1 MgCl_2_, 1 CaCl_2_, 10 HEPES, pH 7.2. The regular pipette solution contained in mM: 145 KCl, 1 MgCl_2_, 10 HEPES, pH 7.2, and 5 EGTA, 3.63 CaCl_2_ – resulting in 0.5 μM free Ca^2+^ as calculated using the Webmaxc extended calculator (http://www.stanford.edu/~cpatton/webmaxcE.htm). Experiments were carried out at room temperature between 20 and 22°C. Sodium hydrosulfide (NaHS, Sigma, Vienna) was used as a source of H_2_S. NaHS at concentrations usually used in the present study did not change the pH of the buffered solution. NaHS solutions prepared shortly before experiments were clear and were usually used 3–5 min but no longer than 20 min. For details of making and working with H_2_S, see Hughes et al. (2009).

Drugs added to the pipette solution: ATP – 1 mM; PKA catalytic subunit 50 units/ml; PKC subunit (PKCsu) 0.1 units/ml; PKC inhibitor fragment 19–31 (PKCin) – 500 nM; PKG – 400 units/ml plus 50 μM cGMP; Staurosporine (STS) – 1 μM; Okadaic Acid (OA) – 100 nM.

### Determination of H_2_S concentrations

NaHS salt dissociates in watery solution to Na^+^ and HS^−^, and HS^−^ associates with H^+^ to produce H_2_S. Previously as a rule of thumb we calculated for neutral solutions that one-third of NaHS exists as H_2_S and the remaining two-thirds are present as HS^−^ (Beauchamp et al., 1984). This provides a solution of H_2_S at a concentration that is about 66% less compared to the original concentration of NaHS.

**Figure 1:**
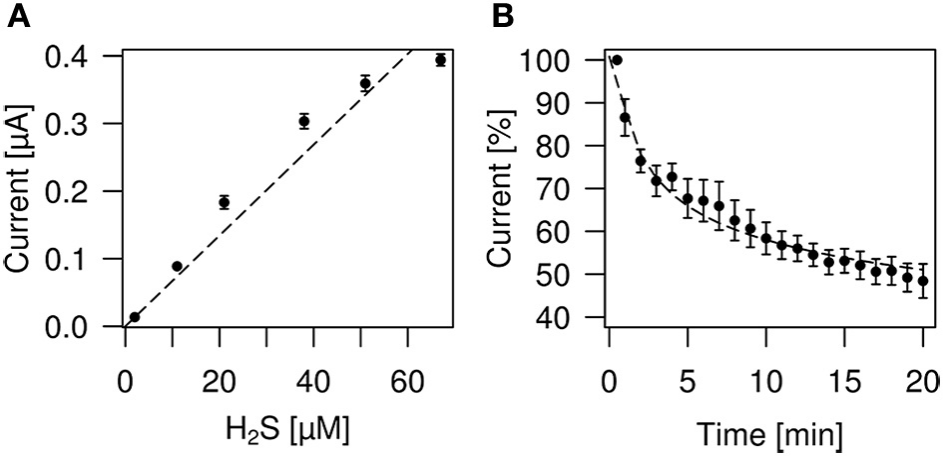
**Determination of H_2_S concentrations and loss of H_2_S by evaporation. (A)** Plot of the H_2_S concentrations derived from NaHS donor vs. H_2_S sensor current (nA). The relationship is linear up to about 50 μM H_2_S, respectively 226 μM NaHS (stippled line fitted to data points). **(B)** Measurement of H_2_S evaporation from physiological bath solution. The plot shows sensor current readings vs. time. One hundred percent indicates the beginning of the measurement which started immediately after preparation of the solution.

In order to obtain more precise measurements for our experimental situation we have determined H_2_S concentrations using an anion selective electrode (ISO-H2S-2) together with an Apollo 1000 free radical Analyser (WPI, Germany). The response time of the sensor is less than 5 s, with a sensitivity of 2 pA/nM and a detection range from 5 nM to 100 μM H_2_S. For calibration of the sensor instructions according to the WPI manual were used. After we had established a calibration curve we determined the rate of loss of H_2_S versus time for the NaHS donor. As depicted from Figure 1B the loss of H_2_S obtained after 20 min is 42%. A loss of about 33% after 15 min and 90% after 30 min was reported previously by Kimura et al. (2006).

The H_2_S sensor measures the dissolved H_2_S gas which is only one component of the total sulfide equilibrium system. The total sulfide concentration [S_*tot*_] in solution can be calculated from: [H_2_S] = [S_tot_]/{1+K_1_/[H^+^] + K_1_K_2_/[H^+^]}, with pK_1_ = 6.89, where K_1_ = 10^−6.89^ and pK_2_ = 19, where K_2_ = 10^−19^ at pH = 7.21, where [H^+^] = 10^−7.21^ M. As K_2_ is very small and not significant at pH < 9 we can use a simplified equation: [H_2_S] = [S_tot_]/(1+K_1_/[H^+^]). For NaHS = 300 μM and pK_1_ = 6.89, the H_2_S concentration calculates to 97 μM, which is quite similar to the rule of thumb of 1/3 H_2_S in solution (see above). Calculated from pK_1_ = 7.04 for standard conditions (20°C, which is close to our experimental conditions) K_1_ = 10^−7.04^ (Lide, 1998). Using the pK1 value of 7.04 and 300 μM of NaHS we calculate 121 μM of H_2_S. However, pK_1_ is also dependent on salinity. The equation derived by Millero (Millero et al., 1988) gives for: pK_1_ = −98.08 + 5765.4/T + 15.04555 × LN(T) + (−0.157 × (S^0.5^)) + 0.0135 × S (with T = temperature in Kelvin, LN(T) = is the natural logarithm of T, and S = salinity). Taken salinity as zero for water and temperature 20°C equal to 293.15 Kelvin we calculate pK_1_ = 7.056, which is very close to literature value (Lide, 1998). The salinity of our extracellular solution is 11.5 gram of salts per liter. Taken this salinity value pK_1_ = 6.679. For 300 μM of NaHS the H_2_S concentration calculates to 68 μM, which is 22.3% of the total sulfide concentration. Hence it is important in addition to pH and temperature to take salinity into account to calculate H_2_S concentrations.

### Calibration

H_2_S liberated from NaHS evaporates quickly from the solution (DeLeon et al., 2012; cf. Olson, 2012). We therefore took measures to avoid loss of H_2_S during calibration or perfusion. A stock solution of 2 mM NaHS was prepared in distilled water or in experimental bath solution in an Eppendorf vial and thoroughly covered using parafilm. 100, 500, 1000, 2000, or 3000 μl of stock solution was added to 20 ml bath solution to obtain the desired NaHS concentration. Figure 1A shows a plot of H_2_S concentrations derived from NaHS donor vs. H_2_S sensor current (nA). The relationship is linear up to about 50 μM H_2_S, respectively 226 μM NaHS. Table 1 shows values of NaHS solutions, the sensor current and the calculated H_2_S concentrations. During dilution from the stock to the final solution which took about 30–60 s we may have lost about 5–10% H_2_S by evaporation (see Figure 1B). The container, holding the NaHS solution had a volume of 20 ml and was covered with parafilm. The fluid was continuously stirred as required for the correct function of the H_2_S sensor using the lowest stirring speed to avoid mechanical perturbation of the sensor.

**Table 1:** **List of NaHS solutions prepared in standard bath solution (first row), the measured sensor current ± standard error of the mean (s.e.m.) (middle row) and the calculated amount of H_2_S (last row)**.

**NaHS, μM**	**Sensor current, nA (±s.e.m.)**	**H_2_S, μM**
10	13.67 ± 0.33	2
48.7	88.67 ± 2.4	11
95.23	183.33 ± 9.61	21
170.2	303.33 ± 11.02	38
226	359.33 ± 11.66	51
300	394.00 ± 8.62	67

From our measurements of H_2_S concentrations we know that during the first 5 min about 30–40% of H_2_S evaporates from the solution (Figure 1). Over the following 5 min, which was the actual application period, about 6–8% H_2_S evaporated. This gives a total of approximately 40–50% loss of H_2_S, i.e., an effective H_2_S concentration during our experimental settings of 11–13%, i.e., 34–41 μM H_2_S from a 300 μM NaHS solution.

### Perfusion settings during recordings

We used a closed perfusion system comprising 5 ml syringes (Henke-Sass, Wolf, HSW, FRG) containing the experimental fluid. The syringe opening was covered with parafilm with a small whole inserted to allow for pressure exchange. The tip of the perfusion pipette was located in the recording chamber at about 1 mm distance to the measured cell or patch. The constant perfusion rate of 1 ml/80 s ensured that the channels recorded were always submerged by the desired H_2_S concentration with little further loss due to evaporation.

### Statistical methods

There are some problematic features of proportional data, like open probabilities (Po), which often hinder the use of parametric statistical procedures, such as ANOVAs and regression analysis. Basic assumptions of these methods are often violated, because all values coming from single channel recordings are set within the interval 0 < Po < 1. To be more precise, proportional data tend to be non-normally distributed and heteroscedastic in nature, which actually means that the size of sample variances is not evenly distributed along the interval of 0 < Po < 1.

Besides using less powerful non-parametric methods, there are other strategies to overcome these limitations, e.g., data transformation (Sokal and Rohlf, 1995) and/or logistic regression. Here, Po-values were transformed with the “logit-function” *p* = ln(p/(1 − p)), which “normalized” this kind of data and substantially reduced heteroscedasticity. After this transformation, “usual” parametric methods could be used. Due to similar reasons, dwell-time measurements were also log-transformed prior to statistical analysis.

Since our experiments also include repeated measurements on similar membrane patches, all statistical procedures had to be adapted accordingly. The number of repetitions of each experiment “n” refers to the number of patches from different cells measured. In electrophysiology, this problem is usually addressed by calculating “fold-changes” of variables, i.e., dividing all measurements of open probabilities with their matched “control values.” Nevertheless, there are also more powerful statistical procedures to handle the problem of self-correlated or repeated measurements, one of them is the use of “mixed models” (Venables and Ripley, 2002; Bolker et al., 2009; Zuur et al., 2009). With this method, not only “fixed factors,” such as experimental conditions, are used but also so called “random factors,” which usually comprise “uncontrolled” variables (Venables and Ripley, 2002; Zuur et al., 2009), such as test subjects, or in this particular case, membrane spots. Since more familiar “*post-hoc* analysis,” such as a “TukeyHSD” or “Scheffe”-test are only feasible in simple univariate ANOVA designs (Sokal and Rohlf, 1995), specific hypothesis tests by linear contrasts had to be used for multiple comparisons (Hothorn et al., 2008). All statistical procedures were performed with R 3.0.1 (R Development Core Team, 2011) and its additional packages “nlme” (Pinheiro et al., 2013) and “multcomp” (Hothorn et al., 2008).

Evaluation of statistic analysis is given as: significant (one star ^*^, 0.05 > *p* > 0.01), high significant (two stars ^**^, 0.01 > *p* > 0.001), and most significant (three stars ^***^, *p* < 0.001). Data described in the text as percentage were calculated by setting control values to 100% and expressing experimental values as percentual change of controls.

## Results

**Figure 2:**
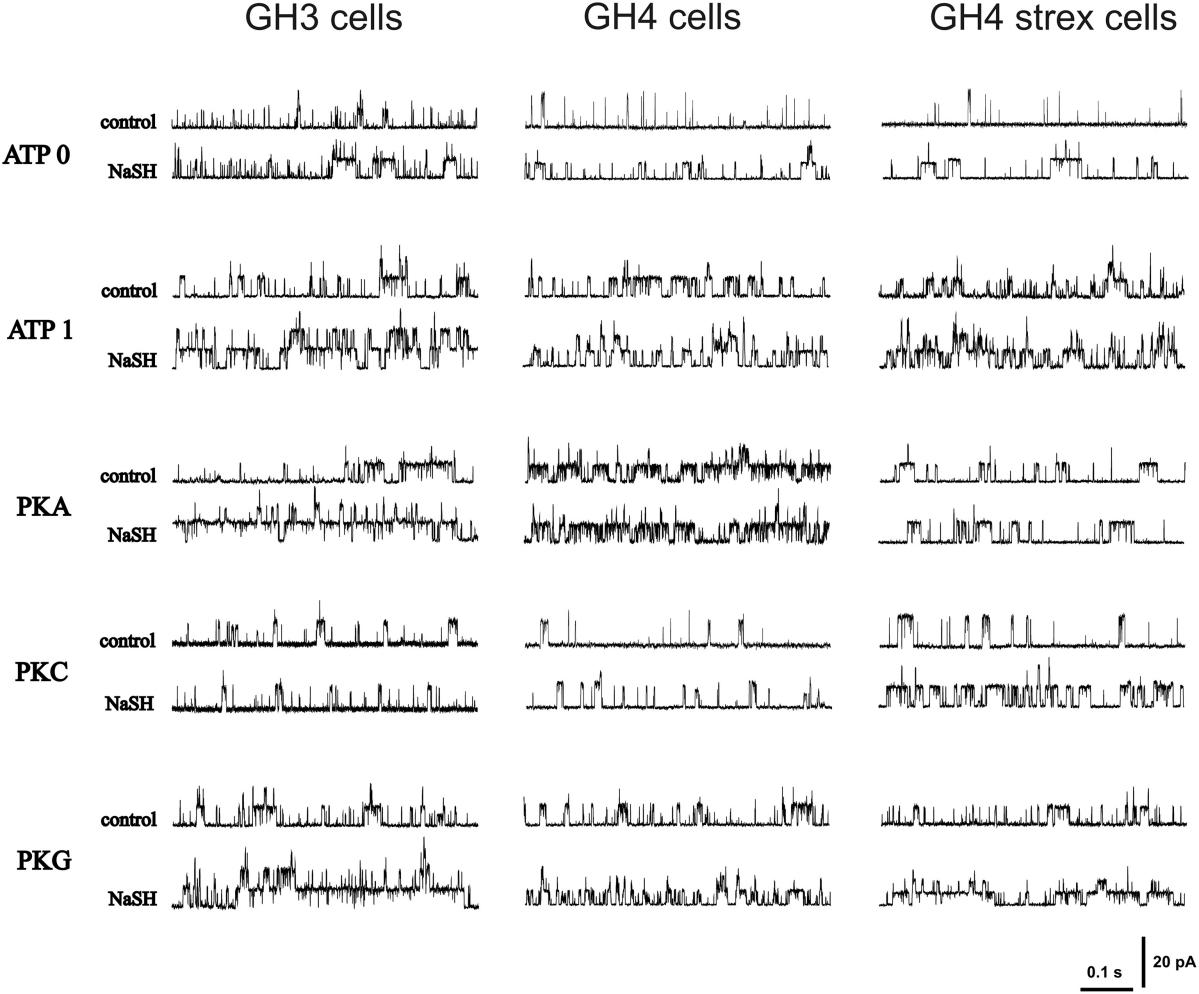
**Original recordings of H_2_S effects on BK single channel open probability (P_open_) during different phosphorylation conditions**. The figure shows recordings from various GH cell types (GH3, GH4, and GH4 STREX) during various phosphorylation conditions (control) and after NaHS (300 μM NaHS, equivalent to effective 34–41 μM H_2_S) application. Holding potential +30 mV.

Original recording of single channel activities are depicted in Figure 2. Patches were held at +30 mV in all recordings.

### Phosphorylation of BK channels

#### GH3 cells

**Figure 3:**
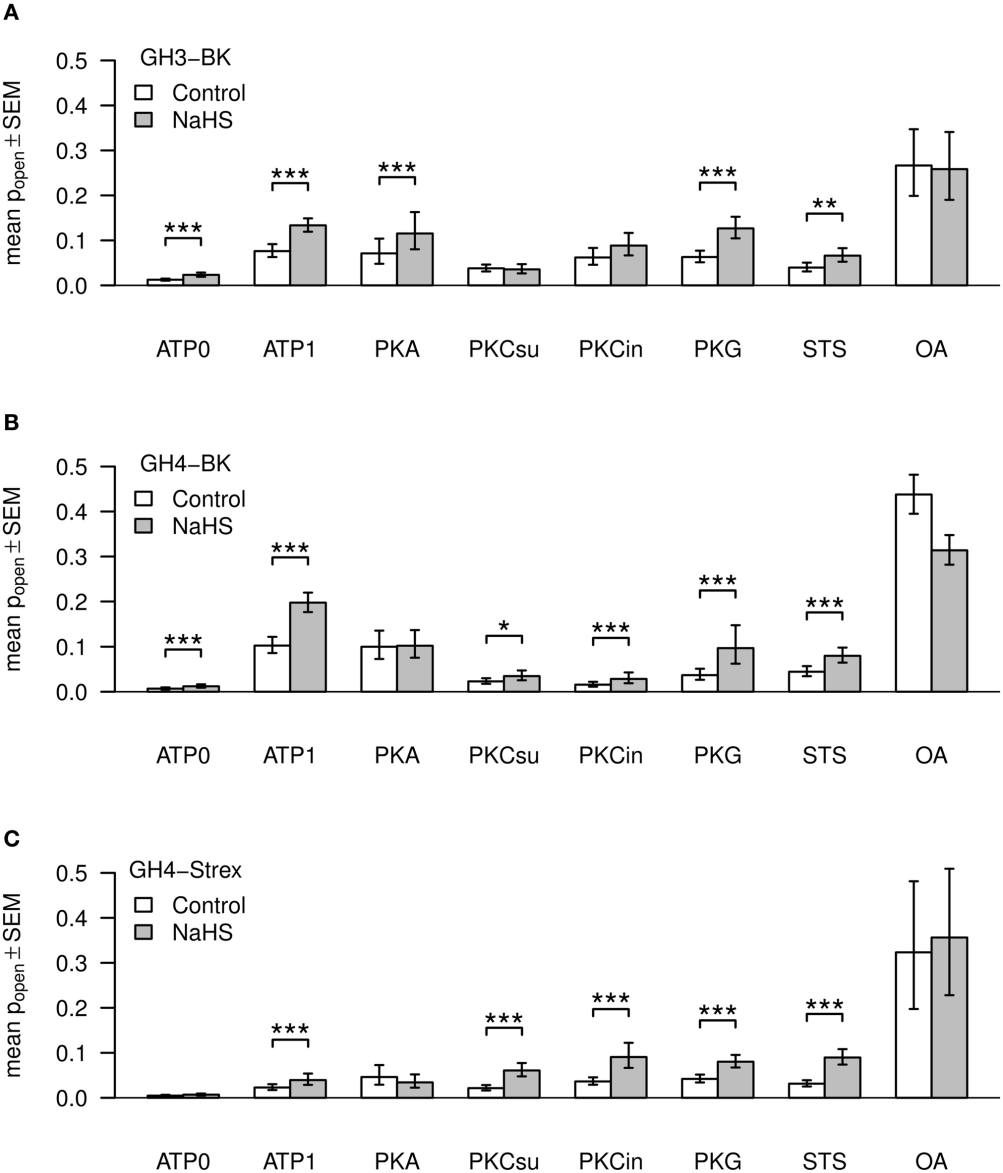
**Mean values of BK channel open time probabilities (P_*open*_)**. Three types of channels, GH3-BK **(A)**, GH4-BK **(B)**, and GH4-STREX **(C)** cells, were investigated. Bar graphs show Po-values under control conditions (left bars, white) and after extracellular application of NaHS (right bars, gray). ATP, kinase, kinase inhibitor, kinase inhibitor staurosporine (STS), or phosphatase blocker okadaic acid (OA) were added to the pipette solution; ATP0 – no ATP added; ATP1 – 1 mM ATP added; PKA (50 units); PKCsu (0.1 units/ml); PKCin – 500 nM; STS – 1 μM; OA – 100 nM. Figures are based on back transformed means and s.e.m. values (error bars), respectively. Asteriks indicate significance of H_2_S (300 μM NaHS equivalent to effective 34–41 μM H_2_S) effects according to the logit transformed data.

Experimental values of BK single channel open probability (Po) for GH3, GH4, and GH4 STREX cells are listed in Table 2. Po-values are presented as mean ± standard error of mean. ATP was added to the pipette solution (intracellular membrane face). In a physiological pipette solution containing no ATP (assigned ATP0), BK single channel open probability (Po) was generally low. Addition of 1 mM ATP (ATP1) to the pipette solution most significantly increased Po (^***^) (Table 2 and Figure 3A). Similar findings have been reported previously from GH3 cells (Denson et al., 2001; Zhou et al., 2012), and reviewed for other cell types (Schubert and Nelson, 2001). All further solutions contained 1 mM ATP. PKA catalytic subunit (50 units/ml) altered Po, but both values were non-significant compared to ATP1 (Figure 3A), however, they were both significantly (^***^) different to ATP0. PKC (PKC catalytic subunit, PKCsu, 0.1 units/ml) in the pipette solution decreased BK Po non-significantly compared to ATP1, but Po was significantly different from ATP0 (^*^). PKC is known to cause inhibition of BK channel activity from GH cells (Shipston and Armstrong, 1996; Hall and Armstrong, 2000; Wu et al., 2007; Zhou et al., 2012, 2010). With protein kinase inhibitor pseudo-substrate (PKCin, fragment 19–31, 500 nM) Po increased non-significantly if compared to PKCsu or ATP1, but was significantly higher than during ATP0 conditions (^**^) if compared to ATP1. The experiments support previous results of an inhibitory role of PKC at BK channels from GH3 cells (Shipston and Armstrong, 1996). Staurosporin (1 μM), an ATP-competitive kinase inhibitor, non-significantly reduced Po, whereas okadaic acid (100 nM), an inhibitor of serine/threonine phosphatase, significantly increased Po compared to 1 mM ATP.

**Table 2:** **Phosphorylation of BK channels**.

	**P_open_**		
	**GH3**	**GH4**	**GH4 STREX**
ATP 0 *n* = 12	0.0152 ± 0.0030	0.0096 ± 0.0028	0.0065 ± 0.0022
ATP 1 *n* = 12	0.0887 ± 0.0135	0.1164 ± 0.0196	0.0342 ± 0.0093
PKA subunit *n* = 11	0.1087 ± 0.0166	0.1256 ± 0.0222	0.0822 ± 0.0321
PKG subunit *n* = 8	0.0724 ± 0.0118	0.0522 ± 0.0173	0.0504 ± 0.0133
PKC subunit *n* = 10	0.0443 ± 0.0075	0.0277 ± 0.0064	0.0253 ± 0.0060
PKC inhib *n* = 6	0.0740 ± 0.0180	0.0208 ± 0.0055	0.0400 ± 0.0087
Staurosporine *n* = 6	0.0468 ± 0.0149	0.0505 ± 0.0147	0.0375 ± 0.0097
Okadaic acid *n* = 7	0.3075 ± 0.0581	0.4422 ± 0.0403	0.3992 ± 0.0962

*Experimental values of BK single channel open probability (P*_open_*) for GH3, GH4, and GH4 STREX cells. ATP0 (no ATP added to the patch pipette); ATP (1 mM added to the patch pipette; all further solutions contained 1 mM ATP during application of PKA, protein kinase A; PKG, protein kinase G; PKC, protein kinase C; STS, staurosporine; OA, okadaic acid; Po-values are presented as mean ± standard error of mean, and n is the number of patches/cells*.

#### GH4/C1 cells

All drug concentrations used for these cells were similar to those used with GH3 cells. With ATP1 Po increased high significantly compared to ATP0 (^***^) (Table 2 and Figure 3B). Using PKA subunit or PKG showed no significant difference to ATP1 containing solution. PKC-subunit reduced Po about 4-fold (^**^) with high significance compared to ATP1, as previously reported by Zhou et al. (2012). Use of PKC inhibitor pseudo-substrate had a similar effect. With the kinase inhibitor staurosporin Po was statistically not significantly different to ATP1 (Table 2, Figure 3B). With the phosphatase inhibitor okadaic acid, however, Po significantly increased if compared to ATP1, PKA, PKCin, PKCsu, staurosporine, or ATP0.

#### GH4/C1 STREX cells

These cells contain a 59 amino-acid insert, so called STREX (stress axis regulated) exon at the BK channel α-subunit C terminus, located between the two regulatory domains of K^+^ conductance (RCK1 and RCK2). This insert bears various cysteine residues which can be phosphorylated. The STREX insert speeds activation and slows deactivation kinetics and their half-activation voltage is shifted to the left (Xie and McCobb, 1998). Again, as in GH3 or GH4/C1 cells Po-values in ATP0 were low (Table 2, Figure 3C), in fact they were the lowest of all three cell types. Po increased with ATP1 with low significance (^*^). The increase caused by ATP was, however, less compared to BK channels from GH3 or GH4 cells (Table 2, Figure 3C). PKA is known to phosphorylate the channels at the STREX insert and changes channel activity from stimulatory to inhibitory whereas channels that lack the STREX insert are activated by PKA (Tian et al., 2001). PKA increased Po significantly when compared with ATP0, but this increase was not significant when compared to ATP1. With PKG Po was not significant different to ATP1. Using PKC-subunit, PKC inhibitor, or staurosporine, again, no significant alteration of Po compared to ATP1 was observed. However, okadaic acid significantly increased Po (^***^) when compared to all other conditions, implying that inhibition of the BK channel attached phosphatase allows for unrestricted phosphorylation of the channel protein.

### H_2_S modulates BK channel activity dependent on phosphorylation

We again present data separately for all three cell types used in our study. Original recordings of H_2_S effects are shown in Figure 2.

#### GH3 cells

As reported previously (Sitdikova et al., 2010) and shown in Figure 3A, NaHS (300 μM – effective H_2_S concentration 34–41 μM, see Materials and Methods), applied to the extracellular bath solution most significantly increased Po of BK channels from 0.01522 ± 0.00302 to 0.0295 ± 0.00722 (*n* = 12, ^***^) (internal ATP free pipette solution (ATP0), *V* = +30 mV), equal to 190.58 ± 13.81% of control (=100%) during the first minute of H_2_S perfusion. Po decayed after 1 min H_2_S application during the following 3 min. A similar transient response was observed previously using ethanol (Jakab et al., 1997) and H_2_S (Sitdikova et al., 2010). In ATP1 solution Po further increased from 0.08872 ± 0.01352 control most significantly to 0.14082 ± 0.01439, *n* = 12 (^***^) with NaHS, i.e., to 191.65 ± 23.52% of control (=100%) (Figure 3A). All further experimental solutions contained 1 mM ATP. Using PKA subunit (50 units/ml), NaHS increased Po with high significance from 0.10873 ± 0.01664 (control) to 0.15475 ± 0.02218, *n* = 11 (^***^), i.e., to 169.42 ± 19.99% of control. PKG (50 units/ml) also increased Po from 0.0724 ± 0.0118 (control) to 0.1395 ± 0.0236(*n* = 8) in NaHS by 205.56 ± 19.08% (^***^). PKC catalytic subunit (PKCsu, 0.1 units/ml) decreased BK channel activity to 0.04434 ± 0.00752 and NaHS had no significant effect (Po = 0.04609 ± 0.00869, *n* = 10, equal to 104.92 ± 16.09%). After addition of PKC inhibitor pseudo-substrate (PKCin, fragment 19–31, 500 nM), Po was 0.07408 ± 0.01808 (control), but NaHS again had no significant effect (Po 0.10292 ± 0.02299, *n* = 6, i.e., to 145.45 ± 14.082%). During application of the kinase inhibitor staurosporine (STS, 1 μM) Po was decreased to 0.04683 ± 0.01493, and in this case NaHS significantly increased Po to 0.07483 ± 0.01892 (*n* = 6, ^**^), i.e., to 168.56 ± 11.87% of control. The most significant increase of Po compared to either ATP0 or ATP1 was obtained with the phosphatase inhibitor okadaic acid (100 nM) which increased Po to 0.3075 ± 0.0581, however, Po was not further significantly altered by NaHS (0.3039 ± 0.05523, *n* = 8, 102.10 ± 13.86%).

#### GH4/C1 cells

All basic conditions and concentrations of drugs were similar to the experiments using GH3 cells. In ATP0, Po increased highly significant from 0.00968 ± 0.00285 (control) to 0.01632 ± 0.00452, *n* = 8 (^***^), i.e., to 183.48 ± 22.12% (^***^) after application of NaHS. With ATP1 solution, Po increased significant from 0.11647 ± 0.01969 (control) to 0.20591 ± 0.02146, in NaHS, *n* = 11 (^***^), i.e., to 201.36 ± 19.12%. After application of PKA, Po was 0.12569 ± 0.02224 (control), but with NaHS no significant alteration of Po 0.12693 ± 0.02414, *n* = 11, i.e., to 107.33 ± 11.86% was observed. With PKG Po increased highly significant from 0.05229 ± 0.01732 (control) to 0.1430 ± 0.04916 (*n* = 7) in NaHS, i.e., by 258.95 ± 33.78% (^***^). Application of PKC subunit (PKCsu, 0.1 units/ml) decreased Po to 0.02771 ± 0.00647 and NaHS increased Po significantly to 0.0465 ± 0.01669, i.e., to 161 ± 28%, *n* = 7 (^*^). A similar Po of 0.02089 ± 0.00553 was obtained with PKC inhibitor (PKCin, 500 nM), and Po was high significantly increased with NaHS to 0.04093 ± 0.0108, *n* = 7 (^***^), i.e., to 186.78 ± 19.85%. With staurosporine (STS, 1 μM) Po was 0.0505 ± 0.01474 and increased with NaHS highly significant to 0.087 ± 0.02195, *n* = 5 (^***^), i.e., to 181.66 ± 15.35%. Okadaic acid (100 nM) substantially increased Po to 0.44223 ± 0.0403 compared to either ATP0 or ATP1 controls, whereas NaHS decreased Po under this condition to 0.31953 ± 0.03142, *n* = 7 (^***^), i.e., to 74.841 ± 7.8%.

#### GH4/C1 STREX cells

In ATP0 BK channel Po of 0.00652 ± 0.00224 (control) was low compared to Po of GH3 and GH4 cells. NaHS non-significantly increased Po to 0.00921 ± 0.00286, *n* = 7, i.e., to 148 ± 11.99%. With ATP1 Po increased to 0.03428 ± 0.00931, *n* = 13 compared to ATP0, but this increase was much less compared to Po-values obtained in GH3 and GH4 cells (Figure 3). NaHS in ATP1 increased Po at high significance to 0.06233 ± 0.01687, *n* = 13 (^***^), i.e., to 177.74 ± 15.91%. PKA subunit (50 units/ml) increased Po to 0.08221 ± 0.03214, *n* = 8 under control conditions, however, NaHS application had no significant effect on Po, 0.05739 ± 0.02129, *n* = 8, i.e., 78.65 ± 9.38% of control. PKG high-significantly increased Po from 0.05043 ± 0.01330 (control) to 0.08829 ± 0.01635 in NaHS, by 201.94 ± 40.37%, *n* = 8 (^***^).

As with previous results from GH3 and GH4 cells, using PKC subunit (PKCsu, 0.1 units/ml) BK channel Po was low, 0.02533 ± 0.00604, *n* = 6 (control), however, NaHS increased Po to 0.06833 ± 0.01267, i.e., to 322.44 ± 74.48%, although with low significance (^*^). With PKC inhibitor (PKCin, 500 nM) Po of 0.0400 ± 0.00879, *n* = 5 was similar to ATP1, and increased with NaHS to 0.10552 ± 0.02854, *n* = 5, i.e., to 253.07 ± 28.82%, however not significantly. Also staurosporine (STS, 1 μM) non-significantly increased Po from 0.0375 ± 0.00975 (control) to 0.10114 ± 0.02397 in NaHS, *n* = 8, i.e., to 306.14 ± 49.76%. In okadaic acid (100 nM) which significantly increased Po to 0.39927 ± 0.09621 compared to ATP1, NaHS had no additional significant effect on Po, 0.42796 ± 0.08584, *n* = 7, i.e., to 114.93 ± 9.22%.

In summary, our result show (Table 3) that Po of BK channels from GH3 and GH4 containing no ATP (ATP0) were high significantly increased in the presence of H_2_S, whereas BK channels from GH4-STREX cell did not respond to H_2_S. Po of BK channels of all cell types phosphorylated by ATP was highly significantly increased and H_2_S further increased Po at high significance. PKA and PKG were not able to increase Po beyond the ATP1 effect. H_2_S was able to increase Po of BK channels from GH3, but not those of GH4 and GH4 STREX cells. H_2_S high significantly increased Po of all cell types pretreated with PKG. PKC-subunit (PKCsu) either had no effect (GH4-STREX) or decreased Po (GH3, GH4) but H_2_S increased Po of BK channels pretreated with PKCsu in GH4 and GH4 STREX but not those of GH3 cells. Staurosporin had no effect on BK channel Po activity but did not prevent the activating effect of H_2_S. Okadaic acid most significantly increased BK channel Po of all cell types and H_2_S had no further effect on channel Po, it even decreased BK channel Po of GH4 cells.

**Table 3:** **Summary, of open probability (Po) of BK channels in the absence (ATP0, first line) and presence of ATP1 (1 mM, all other lines), during application of PKA (protein kinase A), PKG (protein kinase G), PKC (protein kinase C), STS (staurosporine), OA (okadaic acid), and after application of H_2_S**.

**GH3**	**Po**	**GH4**	**Po**	**GH4 Strex**	**Po**
ATP0		ATP0		ATP0	
+H_2_S	+	+H_2_S	+	+H_2_S	0
ATP1	+	ATP 1	+	ATP1	+
+H_2_S	+	+H_2_S	+	+H_2_S	+
PKA	0	PKA	0	P KA	0
+H_2_S	+	+H_2_S	0	+H_2_S	0
PKG	0	PKG	0	PKG	0
+H_2_S	+	+H_2_S	+	+H_2_S	+
PKCsu	−	PKCsu	−	PKCsu	0
+H_2_S	0	+H_2_S	+	+H_2_S	+
STS	0	STS	0	STS	0
+H_2_S	+	+H_2_S	+	+H_2_S	+
OA	+	OA	+	OA	+
+H_2_S	0	+H_2_S	−	+H_2_S	0

*All experimental values were compared to ATP1, except ATP0*.

*+, Po increase; 0, no effect; −, decrease of Po*.

### Channel open dwell times and amplitudes

**Figure 4:**
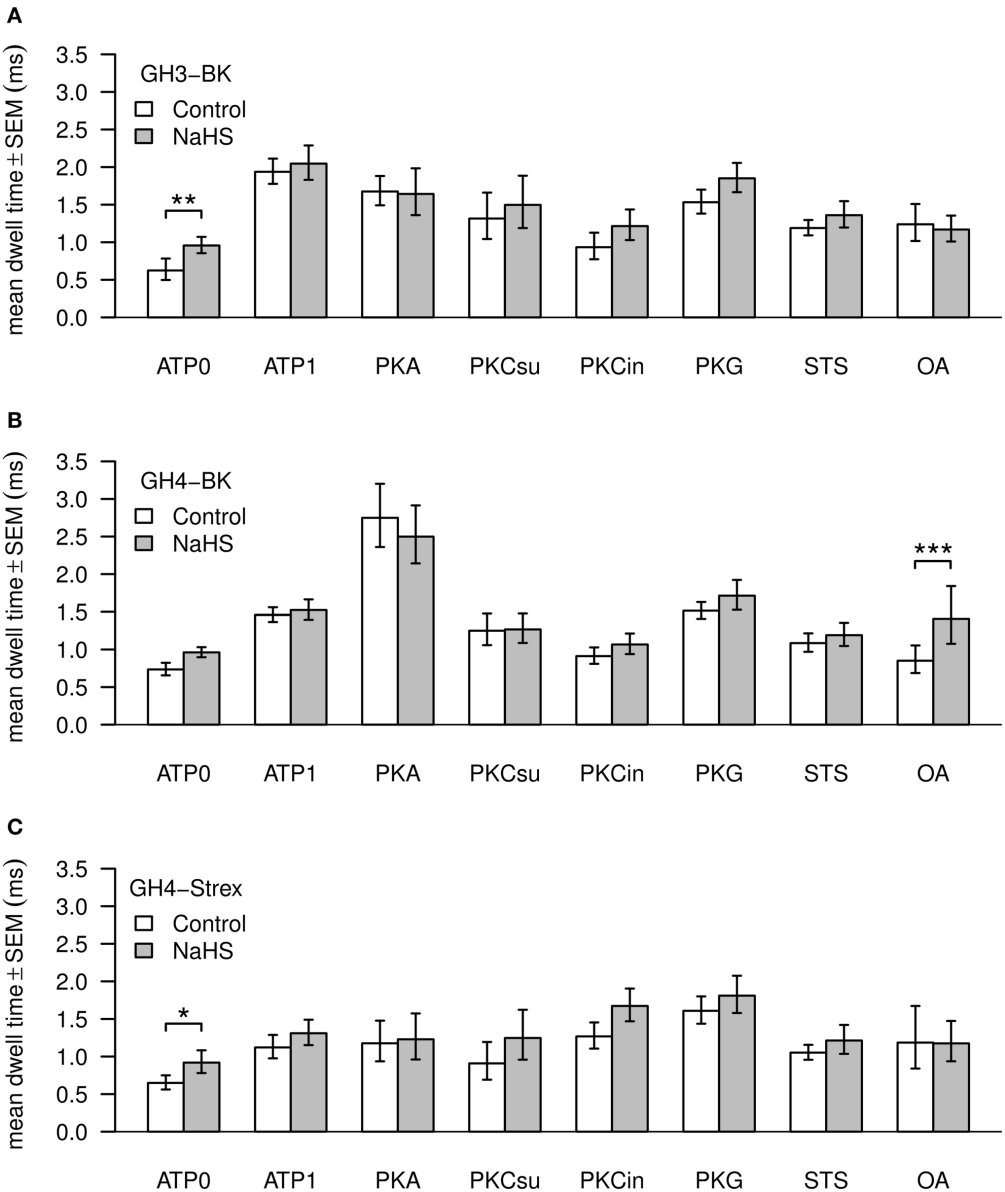
**Mean values of BK channel open dwell times**. Three types of channels—GH3-BK **(A)**, GH4-BK **(B)**, and GH4-STREX **(C)** cells were investigated. Bar graphs show open dwell values in ms under control conditions (left bars, white) and after extracellular application of 300 μM NaHS (right bars, gray). All drug concentrations were the same as shown in Figure 3. Significant ^*^0.05 > *p* > 0.01; high significant ^**^0.01 > *p* > 0.001; most significant ^***^p < 0.001.

**Figure 5:**
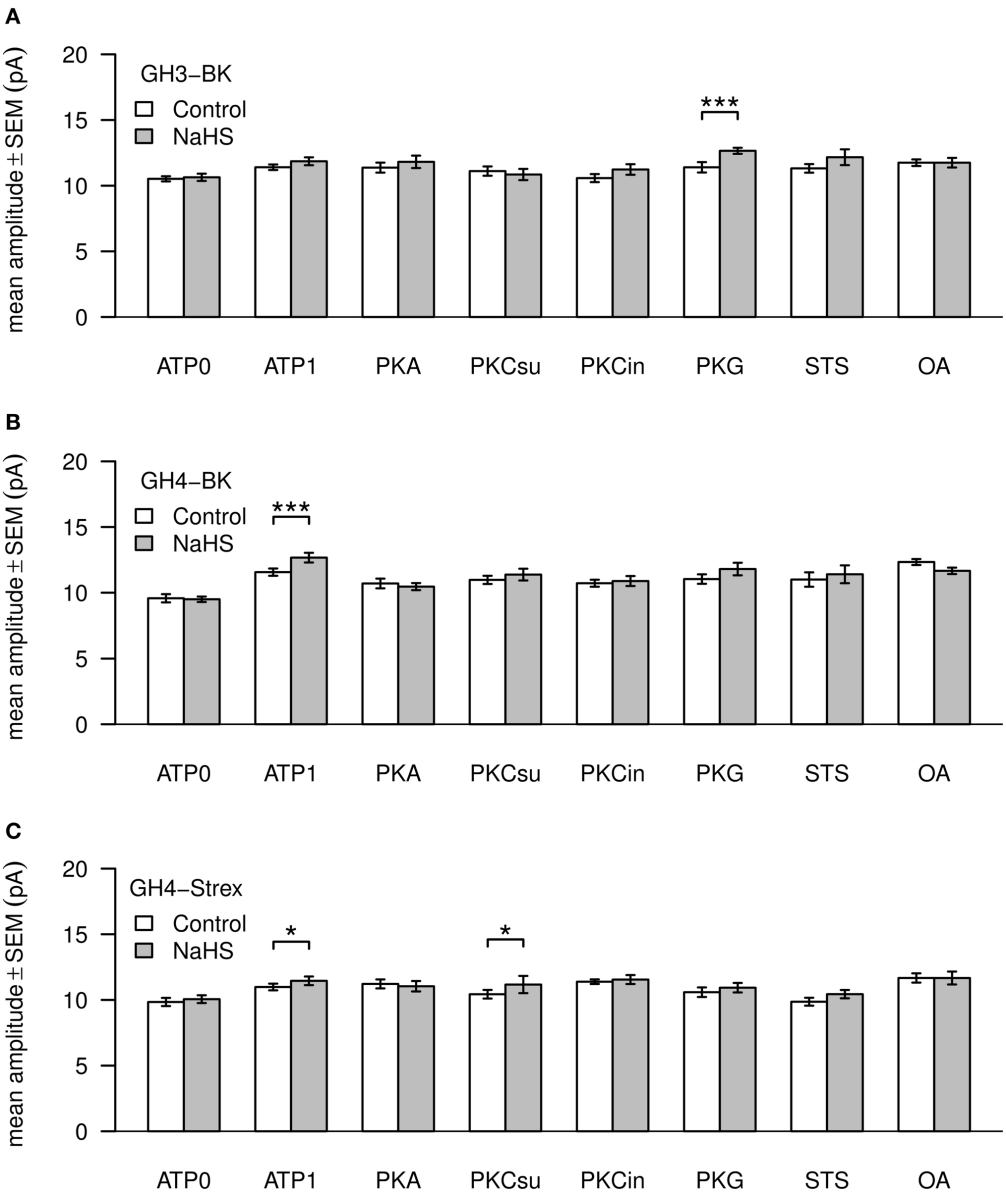
**Mean values of BK channel amplitudes**. Three types of channels—GH3-BK **(A)**, GH4-BK **(B)**, and GH4-STREX **(C)** cells were investigated. Bar graphs show amplitude values in pA under control conditions (left bars, white) and after extracellular application of 300 μM NaHS (right bars, gray). All drug concentrations were the same as depicted in Figure 3. Significant ^*^0.05 > *p* > 0.01; most significant ^***^p < 0.001.

Values of BK channel Po, amplitude and dwell time are shown in Figures 4, 5. Channel open dwell-time indicates the time a channel spends at the open current level. Analysis of BK channels from GH3 cells revealed that ATP most significantly (^***^) increased mean open dwell times from 0.74 ± 0.087 ms (ATP0) to 2.022 ± 0.18 ms (ATP1). Open dwell times were also significantly increased with PKA to 1.78 ± 0.22 ms (^***^), with PKG to 1.56 ± 0.15 ms (^**^) and with PKC subunit to 1.78 ± 0.6 ms (^*^), compared to an ATP0 solution. A significant increase of mean dwell times was also observed with BK channels from GH4 cells from 0.77 ± 0.10 ms (ATP0) to 1.49 ± 0.11 ms (^**^) after application of ATP1. With PKA dwell times increased to 3.00 ± 0.44 ms (^***^) and with PKG to 1.54 ± 0.12 ms (^**^) if compared to ATP1 (Figure 4B). Significant decreases of dwell times were seen with paired comparison for PKCsu vs. PKA (^***^), PKCin vs. PKA (^***^), staurosporine vs. PKA (^***^) and okadaic acid vs. PKA (^***^) and PKG vs. PKA (^*^). NaHS had a significant increasing effect on open dwell times only in okadaic acid (^***^). In GH4 STREX cells open dwell times were only significantly increased when PKG is compared with ATP0 (^*^).

Addition of NaHS to GH3 increased BK channel dwell times significantly only in ATP0 from 0.74 ± 0.087 ms to 1.02 ± 0.11 ms (^**^), with GH4 cells in okadaic acid from 0.10 ± 26 ms to 1.73 ± 0.46 ms (^***^), and in ATP0 in GH4 STREX cells from 0.69 ± 0.10 ms to 0.99 ± 0.16 ms (^*^). NaHS had no effect at dwell times with all other experimental settings (Figure 4C).

Mean amplitudes of single BK channel currents from GH3 cells were not altered under ATP0 or any of the phosphorylation procedures. Only with PKG and H_2_S channel amplitudes were significantly increased (Figure 5A). In GH4 cells channel amplitudes significantly increased in ATP1 vs. ATP0. In all other settings the values could not be differentiated to ATP1 (which was always present in these experiments). H_2_S significantly increased amplitudes only with ATP1 (Figure 5B). Also in GH4 STREX cells BK channels amplitudes were increased at low significance with okadaic acid vs. ATP0 and staurosporin. H_2_S increased channel amplitudes with ATP1 and PKCsu (Figure 5C).

Channel amplitudes and open dwell time histograms are listed as Supplementary Material.

## Discussion

### Recalculation of H_2_S concentrations

Experimentally used H_2_S concentrations are of major concern in the context of physiological quantities. In the present study we recalculated the concentration of H_2_S liberated from the donor NaHS. In our calculations and estimations we were taking in addition to temperature and pH the salinity of the solution and evaporation of H_2_S into account. The results indicate that a concentration of 300 μM NaHS, which was usually used in our experiments (Sitdikova et al., 2010) contains approximately 68 μM H_2_S, i.e., 22.3% of the NaHS concentration is as H_2_S in solution. Furthermore, taking evaporation of H_2_S into account we estimated a loss of approximately 40–50% of H_2_S which gives an *effective* H_2_S concentration during our experimental settings of 11–13%, i.e., 34–41 μM H_2_S from a 300 μM NaHS solution.

Our previous estimations of the effective NaHS concentrations (EC50) at BK channel Po (Sitdikova et al., 2010) gave a low range value of 169 μM and a high range value of 2000 μM. With the newly calculated values we obtain for the low range NaHS value an EC50 of 18.8–22.6μM, for the high range EC50 223–267.6 μM of actual H_2_S in solution. Early estimations of the amount of H_2_S concentrations in the rat, bovine, and human brain gave values of 50–160 μM (Goodwin et al., 1989; Warenycia et al., 1989; Savage and Gould, 1990). These values appear overestimated since with the method used in these studies H_2_S from acid-labile sulfur was measured in addition to free H_2_S (Kimura et al., 2012). Some recent experimental H_2_S estimations suggest extremely low values of basal submicromolar concentrations increasing to single-digit micromolar levels during application of cysteine or hypercapnia (Leffler et al., 2011). In our studies we usually used a standard concentration of 300 μM NaHS (34–41 μM effective H_2_S) to (1) obtain reliable, well visible effects at the channels, and (2) being able to compare our results with previous investigations. Previous studies (Sitdikova et al., 2010) indicated that the lowest H_2_S concentration that significantly (*p* < 0.05) increased Po was about 30 μM NaHS, which effectively amounts to 3.4–4.1 μM free H_2_S. Hence, H_2_S appears to induce BK channel activation at a physiological relevant concentration range. The effective H_2_S level at its target site(s) is unknown, however. H_2_S may be stored as bound sulfane sulfur and/or neuronal excitation can trigger its release from surrounding astrocytes (Ishigami et al., 2009). It may be possible that subcellular local high concentrations of H_2_S may be effective at ion channels for a short period of time which is supported by the fact that even very high concentrations at BK channels (400–500 μM) applied for a few minutes were completely reversible (Sitdikova et al., 2010). Therefore, not only the amount of H_2_S appears important but the concentration per application time.

Our results indicate that the effect of NaHS on single channel currents was maximal within 1 min of application but then decreased within the following minutes. The decline of Po may be caused by the evaporation of H_2_S from the perfusate. This appears unlikely, however, since the decline of H_2_S within the 5 minute actual experimental time (see Materials and method) was only about 6–8%. As indicated from a previous dose-response plot (Sitdikova et al., 2010, Figure 4), this will result in a Po variation within the experimental error and therefore is unable to explain the decrease of Po. And, most importantly—we continuously perfuse the patches which gives a constant concentration of H_2_S at the channels. We therefore interpret this transient response of the BK Po as development of tolerance to the gas. A similar transient effect has been reported for the action of ethanol on BK channels from GH3 cells (Jakab et al., 1997; Treistman and Martin, 2009). The transient activation of ion channels may be physiologically relevant since this mechanism would allow cells to readjust to pre-stimulation electrical signaling conditions even in the presence of high levels of the activating drug or it may play a role in the rapid adaptation of other excitable cells, such as receptor cells.

In physiological solutions at pH 7.4, H_2_S (~20%), HS^−^ (~80%), and traces of S^2−^, are always simultaneously present in the solution or in the cytosol. This mixture is usually referred to in the literature as H_2_S for simplicity (Liu et al., 2012; Greiner et al., 2013). HS^−^ should not be able to cross the membrane since it is charged and negative surface charges at the membrane make a passage unlikely. H_2_S, as a neutral molecule appears more likely to readily cross the membrane. An internal action of H_2_S at BK channels appears indicated since the effect of NaHS was prevented when the reducing agent dithiothreitol was applied inside through the pipette of excised outside-out patches, whereas the oxidizing agent thimerosal increased Po. Added to the external solution these agents had no effect (Sitdikova et al., 2010). The experiment suggests that H_2_S is able to penetrate the membrane and acts on an internal site at the channels. Since H_2_S once inside the cell is able to dissociate HS^−^ may be present again. Hence, it is still not clear which, H_2_S or HS^−^, or both may be considered as acting agents. However, an inner ring of negative charges produced by the RCK1,2 C-terminal part of the channel protein (Carvacho et al., 2008) may prevent access of HS^−^ to the channel favoring H_2_S as the acting molecule.

To further complicate the H_2_S issue, recent studies indicate that whatever form of H_2_S donor or even H_2_S itself is used in solutions results in the formation of polysulfides (Greiner et al., 2013) and polysulfides activating TRP receptors are orders of magnitude more effective compared to H_2_S donors (Kimura et al., 2013). Since NaHS contains polysulfides it is possible that at least parts of the results are mediated by polysulfides. Further experimentation is needed to investigate these questions.

Considering the physiological relevance of our findings we speculate that the activation of BK channels causes a decrease of excitability or shorten the duration of action potentials which may lead to an increase of excitability which is modulated by the status of channels phosphorylation. Again, further experimentation is required.

### Phosphorylation and H_2_S effects

ATP with high significance increased Po of all three types of cells investigated, GH3, GH4/C1, and GH4/C1 STREX by 583, 1203, and 525%, respectively, compared to ATP0. Kinases and phosphatases which are constitutively attached to BK channels, arranged in so called nano-domains, appear to provide an equilibrium of phosphorylation/dephosphorylation dependent on the presence of ATP. The most significant effect of ATP observed with GH4 cells suggests that they are particularly amenable to phosphorylation. As to the impact of ATP on BK channels it has been reported that ATP per see can inhibit BK channel activity (Clark et al., 1999; Hirano et al., 2001). This, however, appears not to apply to our situation since ATP in all BK channels from the different cell types investigated increased channel activity. It may be also possible that channel phosphatases are blocked by an ATP-dependent mechanism which could explain the substantial stimulatory effect of ATP. In GH4/C1 cells BK channels were found to be activated by PKG and inhibited by PKA and PKC (White et al., 1991; Shipston and Armstrong, 1996), but this can be altered by PKC-dependent pre-phosphorylation (Zhou et al., 2001). Stimulation of BK channels by ATP, therefore, may depend on the preexisting status of phosphorylation before ATP is supplied.

With pipette solutions containing no ATP H_2_S significantly increased Po of GH3 and GH4 cells but had no significant effect on GH4 STREX cells. This may be explained by the particular arrangement at BK STREX channels where the 59 amino acid insert may prevent access of H_2_S to its activation site. H_2_S application to the bath in ATP-containing pipette solutions significantly increased Po in all cell types. The experiment indicates that phosphorylation (or an ATP/kinase activated blockade of a phosphatase) which in turn may prime the channels for H_2_S activation.

In all three cell lines PKA effect on Po is not significant different compared to ATP1. The result indicates that the channels are already sufficiently phosphorylated in the presence of ATP. PKA has been reported to activate, inhibit or have no effect on BK channels as reviewed by Schubert and Nelson (2001). These different actions appear to depend on alternative BK splicing or could reflect variants in α and β subunit composition of the channel complexes (Hall and Armstrong, 2000; Tian et al., 2001, 2004). At PKA primed channels H_2_S only had a high significant effect on BK channel Po from GH3 cells but had no effect on GH4 and GH4 STREX cells. Phosphorylation of BK channels by PKA therefore appears to prevent further H_2_S activation in these cells. PKG had no significant effect on Po compared to ATP1 of all cell types (GH3, GH4, and GH4 STREX cells), whereas NaHS significantly increased Po of BK channels phosphorylated by PKG of all cell types. In all of our experiments the initial phosphorylation state of BK channels at the start of experiments was not determined and remains unknown. Also the amount of phosphorylation by ATP or the addition of kinases was not measured. This should be kept in mind when considering the interpretation of the results.

PKC subunit and PKC inhibitor decrease channel Po but this effect was only significant with GH4 cells. Our data are in congruence with a decrease of channel activity by PKC reported previously (Shipston and Armstrong, 1996; Schubert and Nelson, 2001; Tian et al., 2004; Kizub et al., 2010; Zhou et al., 2010, 2012; van Welie and du Lac, 2011). H_2_S in this context did not alter BK channel Po from GH3 cells, but increased Po of GH4 and even more of GH4 STREX cells. With PKC inhibitor, H_2_S had no effect at GH3 cells but high significantly increased Po of BK channels from GH4 and GH4 STREX cells. Although PKC phosphorylation suppresses Po of BK channels H_2_S appears able to overcome this effect and to stimulate the channels. The kinase inhibitor, staurosporine, had no significant effect on BK channel Po of all three cell lines. H_2_S under these conditions was able, however, to significantly increase Po. Hence, in all three cell types Po of non-phosphorylated (ATP0) and phosphorylated channels by ATP1 and by PKG can be elevated by H_2_S. PKC phosphorylation prevented the Po increasing H_2_S effect in GH3 cells but increased Po in GH4 and GH4 STREX cells. PKA also differently affected cell types with increasing Po of GH3 cells but not of GH4 and GH4 STREX cells (which appear even decreased by H_2_S).

The protein phosphatase inhibitor, okadaic acid, had the most significant effect in increasing Po under normal conditions (pipette solution containing 1 mM ATP). This peculiar action of okadaic acid may be due to the fact that a very active protein phosphatase is associated with the BK channels (Reinhart and Levitan, 1995; Zhou et al., 2010) and on site acts by dephosphorylating the channels. Inhibition of dephosphorylation by okadaic acid promotes phosphorylation and therefore appears able to convey a major impact on Po activation. Although phosphorylated channels are activated by H_2_S okadaic acid had no further increasing effect on BK channels from GH3 and GH4 STREX cells. We interpret this result by assuming that channel phosphorylation may be at its maximum and therefore H_2_S had no further effect.

The PKC subunit tends to decrease Po but this was only significant with channels from GH4 cells. In the presence of PKC H_2_S increased channel Po of GH4 and GH4 STREX significantly but not those of GH3 cells. The PKC inhibitor had no significant effect on Po compared to PKC subunit, whereas H_2_S had a similar increasing effect in GH4 and GH4 STREX cells. No significant difference was observed between ATP1 and staurosporine, but in all cells channel Po was increased during H_2_S application. Our results indicate that H_2_S modulates diverse BK splice variants in a different way.

A significant increase in open dwell times was observed with ATP1 vs. ATP0 in GH3 cells, in all other settings there was no significant effect beyond the ATP1 increase. In GH3 cells H_2_S only increased open dwell times in ATP0 significantly. With GH4 cells open dwell times increased in ATP1 vs. ATP0 and with ATP1 vs. PKA, but decreased with PKC, PKG, staurosporin, and okadaic acid vs. PKA. A significant increase of dwell times was seen with H_2_S in the presence of okadaic acid. With GH4 STREX cells a low significant increase of dwell times was only observed with PKG vs. ATP0 and with H_2_S in ATP0. Closed dwell times were not analyzed since most of our single channels recording contained more than one channel.

BK channel amplitudes were little altered by phosphorylation or by H_2_S. Exceptions appear to be BK channels from GH3 and GH4 cells which increase in amplitude significantly with PKG or with ATP1, respectively. The mechanisms underlying these alteration in Po, dwell times or amplitude variations caused by phosphorylation of channels and the impact by H_2_S are unknown.

In summary, Po of BK channels of GH3, GH4 cell types, either in their non-phosphorylated state (with no ATP or with staurosporine in the pipette solution) or if phosphorylated by ATP can be activated by H_2_S. GH4 STREX BK channels do not respond to H_2_S in ATP0 whereas in the phosphorylated mode (ATP1) channel Po is increased. PKA prevented the action of H_2_S on channel Po in GH4 and GH4 STREX but increased BK Po of GH3 cells at high significance. This suggests that PKA phosphorylates the channels of GH4 and GH4 STREX cells at some other site which rearranges the protein in a way that H_2_S is no longer able to act at the channels. Zhou et al. (2012) showed that only BK channels which are dephosphorylated as well as depalmitoylated can be stimulated by PKA whereas if phosphorylated by PKC and palmitoylated the channels are insensitive to PKA. PKG vs. ATP0 increased Po of BK channels of all cell types but had no further effect at Po if ATP was present. H_2_S, high significantly increased Po of PKG pretreated cells. This suggests that the phosphorylation by PKG primes the channels for H_2_S activation. PKC decreased Po under control conditions of all BK channels types but BK channel Po of GH4 and GH4 STREX cells was increased with H_2_S at high significance. In experiments using the phosphatase inhibitor okadaic acid, the effect of H_2_S on Po was prevented or even reduced indicating that either phosphorylation at the BK channel protein mediates this effect or the channel activity is already at a maximum preventing any further increase (Table 3).

Our experimental results indicate that phosphorylation may prime or prevent the action of H_2_S on BK channels Po which may be dependent on the channels conformation which exposes or impedes phosphorylation sites and this way may govern the access of H_2_S to reach its effective location.

### Conflict of interest statement

The authors declare that the research was conducted in the absence of any commercial or financial relationships that could be construed as a potential conflict of interest.

## References

[B1] Abe K. Kimura H. (1996). The possible role of hydrogen sulfide as an endogenous neuromodulator. J. Neurosci 16, 1066–1071. 8558235 10.1523/JNEUROSCI.16-03-01066.1996 PMC6578817

[B2] Alioua A. Tanaka Y. Wallner M. Hofmann F. Ruth P. Meera P.. (1998). The large conductance, voltage-dependent, and calcium-sensitive K+ channel, Hslo, is a target of cGMP-dependent protein kinase phosphorylation *in vivo*. J. Biol. Chem. 273, 32950–32956. 10.1074/jbc.273.49.32950 9830046

[B3] Barman S. A. Zhu S. White R. E. (2004). PKC activates BKCa channels in rat pulmonary arterial smooth muscle via cGMP-dependent protein kinase. Am. J. Physiol. Lung Cell. Mol. Physiol. 286, L1275–L1281. 14966080 10.1152/ajplung.00259.2003

[B4] Beauchamp R. O. Jr. Bus J. S. Popp J. A. Boreiko C. J. Andjelkovich D. A. (1984). A critical review of the literature on hydrogen sulfide toxicity. Crit. Rev. Toxicol. 13, 25–97. 10.3109/10408448409029321 6378532

[B5] Berkefeld H. Fakler B. Schulte U. (2010). Ca2+-activated K+ channels: from protein complexes to function. Physiol. Rev. 90, 1437–1459. 10.1152/physrev.00049.2009 20959620

[B6] Bolker B. M. Brooks M. E. Clark C. J. Geange S. W. Poulsen J. R. Stevens M. H. H.. (2009). Generalized linear mixed models: a practical guide for ecology and evolution. Trends Ecol. Evol. (Amst.) 24, 127–135. 10.1016/j.tree.2008.10.008 19185386

[B7] Carvacho I. Gonzalez W. Torres Y. P. Brauchi S. Alvarez O. Gonzalez-Nilo F. D.. (2008). Intrinsic electrostatic potential in the BK channel pore: role in determining single channel conductance and block. J. Gen. Physiol. 131, 147–161. 10.1085/jgp.200709862 18227273 PMC2213566

[B8] Clark A. G. Hall S. K. Shipston M. J. (1999). ATP inhibition of a mouse brain large-conductance K+ (mslo) channel variant by a mechanism independent of protein phosphorylation. J. Physiol. (Lond.) 516(Pt 1), 45–53. 10.1111/j.1469-7793.1999.045aa.x 10066921 PMC2269205

[B9] Cui J. (2010). BK-type calcium-activated potassium channels: coupling of metal ions and voltage sensing. J. Physiol. (Lond.) 588, 4651–4658. 10.1113/jphysiol.2010.194514 20660558 PMC3010134

[B10] Cui J. Yang H. Lee U. S. (2009). Molecular mechanisms of BK channel activation. Cell. Mol. Life Sci. 66, 852–875. 10.1007/s00018-008-8609-x 19099186 PMC2694844

[B11] Dai S. Hall D. D. Hell J. W. (2009). Supramolecular assemblies and localized regulation of voltage-gated ion channels. Physiol. Rev. 89, 411–452. 10.1152/physrev.00029.2007 19342611 PMC2733249

[B12] DeLeon E. R. Stoy G. F. Olson K. R. (2012). Passive loss of hydrogen sulfide in biological experiments. Anal. Biochem. 421, 203–207. 10.1016/j.ab.2011.10.016 22056407

[B13] Denson D. D. Wang X. Worrell R. T. AlKhalili O. Eaton D. C. (2001). Cytosolic phospholipase A2 is required for optimal ATP activation of BK channels in GH(3) cells. J. Biol. Chem. 276, 7136–7142. 10.1074/jbc.M009566200 11113145

[B14] Erxleben C. Everhart A. L. Romeo C. Florance H. Bauer M. B. Alcorta D. A.. (2002). Interacting effects of N-terminal variation and strex exon splicing on slo potassium channel regulation by calcium, phosphorylation, and oxidation. J. Biol. Chem. 277, 27045–27052. 10.1074/jbc.M203087200 12016222

[B15] Furne J. Saeed A. Levitt M. D. (2008). Whole tissue hydrogen sulfide concentrations are orders of magnitude lower than presently accepted values. Am. J. Physiol. Regul. Integr. Comp. Physiol. 295, R1479–R1485. 10.1152/ajpregu.90566.2008 18799635

[B16] Gadalla M. M. Snyder S. H. (2010). Hydrogen sulfide as a gasotransmitter. J. Neurochem. 113, 14–26. 10.1111/j.1471-4159.2010.06580.x 20067586 PMC2965526

[B17] García-Bereguiaín M. A. Samhan-Arias A. K. Martín-Romero F. J. Gutiérrez-Merino C. (2008). Hydrogen sulfide raises cytosolic calcium in neurons through activation of L-type Ca2+ channels. Antioxid. Redox Signal. 10, 31–42. 10.1089/ars.2007.1656 17956188

[B18] Ghatta S. Nimmagadda D. Xu X. O'Rourke S. T. (2006). Large-conductance, calcium-activated potassium channels: structural and functional implications. Pharmacol. Ther. 110, 103–116. 10.1016/j.pharmthera.2005.10.007 16356551

[B19] Goodwin L. R. Francom D. Dieken F. P. Taylor J. D. Warenycia M. W. Reiffenstein R. J.. (1989). Determination of sulfide in brain tissue by gas dialysis/ion chromatography: postmortem studies and two case reports. J. Anal. Toxicol. 13, 105–109. 10.1093/jat/13.2.105 2733387

[B20] Greiner R. Pálinkás Z. Bäsell K. Becher D. Antelmann H. Nagy P.. (2013). Polysulfides link H2S to protein thiol oxidation. Antioxid. Redox Signal. 19, 1749–1765. 10.1089/ars.2012.5041 23646934 PMC3837443

[B21] Grimm P. R. Sansom S. C. (2010). BK channels and a new form of hypertension. Kidney Int. 78, 956–962. 10.1038/ki.2010.272 20720523 PMC3134256

[B22] Hall S. K. Armstrong D. L. (2000). Conditional and unconditional inhibition of calcium-activated potassium channels by reversible protein phosphorylation. J. Biol. Chem. 275, 3749–3754. 10.1074/jbc.275.6.3749 10660522

[B23] Hermann A. Sitdikova G. F. Weiger T. M. (2012a). BK channels – focus on polyamines, ethanol/acetaldehyde and hydrogen sulfide (H2S), in Patch Clamp Technique, ed Shad Kaneez F. (InTech). Available online at: http://www.intechopen.com/statistics/33631 (Accessed September 20, 2013).

[B24] Hermann A. Sitdikova G. F. Weiger T. M. (2012b). Gasotransmitters: Physiology and Pathophysiology. Berlin; Heidelberg: Springer Available online at: http://www.springer.com/biomed/human+physiology/book/978-3-642-30337-1 (Accessed September 27, 2013).

[B25] Hermann A. Sitdikova G. F. Weiger T. M. (2012c). Modulated by gasotransmitters: BK channels, in Gasotransmitters: Physiology and Pathophysiology, eds Hermann A. Sitdikova G. F. Weiger T. M. (Berlin; Heidelberg: Springer), 163–201 Available online at: http://link.springer.com/chapter/10.1007/978-3-642-30338-8_6 (Accessed September 20, 2013).

[B26] Hill M. A. Yang Y. Ella S. R. Davis M. J. Braun A. P. (2010). Large conductance, Ca2+-activated K+ channels (BKCa) and arteriolar myogenic signaling. FEBS Lett. 584, 2033–2042. 10.1016/j.febslet.2010.02.045 20178789 PMC3017811

[B27] Hirano J. Nakamura K. Kubokawa M. (2001). Properties of a Ca(2+)-activated large conductance K(+) channel with ATP sensitivity in human renal proximal tubule cells. Jpn. J. Physiol. 51, 481–489. 10.2170/jjphysiol.51.481 11564285

[B28] Hothorn T. Bretz F. Westfall P. (2008). Simultaneous inference in general parametric models. Biom. J. 50, 346–363. 10.1002/bimj.200810425 18481363

[B29] Hou S. Heinemann S. H. Hoshi T. (2009). Modulation of BKCa channel gating by endogenous signaling molecules. Physiology (Bethesda) 24, 26–35. 10.1152/physiol.00032.2008 19196649 PMC2914466

[B30] Hu L.-F. Lu M. Hon Wong P. T. Bian J.-S. (2011). Hydrogen sulfide: neurophysiology and neuropathology. Antioxid. Redox Signal. 15, 405–419. 10.1089/ars.2010.3517 20812864

[B31] Hughes M. N. Centelles M. N. Moore K. P. (2009). Making and working with hydrogen sulfide: the chemistry and generation of hydrogen sulfide *in vitro* and its measurement *in vivo*: a review. Free Radic. Biol. Med. 47, 1346–1353. 10.1016/j.freeradbiomed.2009.09.018 19770036

[B32] Ishigami M. Hiraki K. Umemura K. Ogasawara Y. Ishii K. Kimura H. (2009). A source of hydrogen sulfide and a mechanism of its release in the brain. Antioxid. Redox Signal. 11, 205–214. 10.1089/ars.2008.2132 18754702

[B33] Jackson-Weaver O. Paredes D. A. Gonzalez Bosc L. V. Walker B. R. Kanagy N. L. (2011). Intermittent hypoxia in rats increases myogenic tone through loss of hydrogen sulfide activation of large-conductance Ca(2+)-activated potassium channels. Circ. Res. 108, 1439–1447. 10.1161/CIRCRESAHA.110.228999 21512160 PMC3234884

[B34] Jakab M. Weiger T. M. Hermann A. (1997). Ethanol activates maxi Ca2+-activated K+ channels of clonal pituitary (GH3) cells. J. Membr. Biol. 157, 237–245. 10.1007/PL00005895 9178611

[B35] Jiang B. Tang G. Cao K. Wu L. Wang R. (2010). Molecular mechanism for H(2)S-induced activation of K(ATP) channels. Antioxid. Redox Signal. 12, 1167–1178. 10.1089/ars.2009.2894 19769462

[B36] Kabil O. Motl N. Banerjee R. (2014). H2S and its role in redox signaling. Biochim. Biophys. Acta 1844, 1355–1366. 10.1016/j.bbapap.2014.01.002 24418393 PMC4048824

[B37] Kawabata A. Ishiki T. Nagasawa K. Yoshida S. Maeda Y. Takahashi T.. (2007). Hydrogen sulfide as a novel nociceptive messenger. Pain 132, 74–81. 10.1016/j.pain.2007.01.026 17346888

[B38] Kim J.-Y. Park C.-S. (2008). Potentiation of large-conductance calcium-activated potassium (BK(Ca)) channels by a specific isoform of protein kinase C. Biochem. Biophys. Res. Commun. 365, 459–465. 10.1016/j.bbrc.2007.10.179 17991423

[B39] Kimura H. (2011). Hydrogen sulfide: its production and functions. Exp. Physiol. 96, 833–835. 21527544 10.1113/expphysiol.2011.057455

[B39a] Kimura Y. Dargusch R. Schubert D. Kimura H. (2006). Hydrogen sulfide protects HT22 neuronal cells from oxidative stress. Antioxid. Redox Signal. 8, 661–670. 10.1089/ars.2006.8.661 16677109

[B39b] Kimura Y. Kimura H. (2004). Hydrogen sulfide protects neurons from oxidative stress. FASEB J. 18, 1165–1167. 10.1096/fj.04-1815fje 15155563

[B40] Kimura H. Shibuya N. Kimura Y. (2012). Hydrogen sulfide is a signaling molecule and a cytoprotectant. Antioxid. Redox Signal. 17, 45–57. 10.1089/ars.2011.4345 22229673 PMC3342561

[B41] Kimura Y. Goto Y.-I. Kimura H. (2010). Hydrogen sulfide increases glutathione production and suppresses oxidative stress in mitochondria. Antioxid. Redox Signal. 12, 1–13. 10.1089/ars.2008.2282 19852698

[B42] Kimura Y. Mikami Y. Osumi K. Tsugane M. Oka J. Kimura H. (2013). Polysulfides are possible H2S-derived signaling molecules in rat brain. FASEB J. 27, 2451–2457. 10.1096/fj.12-226415 23413359

[B43] Kizub I. V. Pavlova O. O. Ivanova I. V. Soloviev A. I. (2010). Protein kinase C-dependent inhibition of BK(Ca) current in rat aorta smooth muscle cells following gamma-irradiation. Int. J. Radiat. Biol. 86, 291–299. 10.3109/09553000903564042 20353339

[B44] Kyle B. D. Hurst S. Swayze R. D. Sheng J. Braun A. P. (2013). Specific phosphorylation sites underlie the stimulation of a large conductance, Ca(2+)-activated K(+) channel by cGMP-dependent protein kinase. FASEB J. 27, 2027–2038. 10.1096/fj.12-223669 23407708

[B45] Lee U. S. Cui J. (2010). BK channel activation: structural and functional insights. Trends Neurosci. 33, 415–423. 10.1016/j.tins.2010.06.004 20663573 PMC2929326

[B46] Leffler C. W. Parfenova H. Basuroy S. Jaggar J. H. Umstot E. S. Fedinec A. L. (2011). Hydrogen sulfide and cerebral microvascular tone in newborn pigs. Am. J. Physiol. Heart Circ. Physiol. 300, H440–H447. 10.1152/ajpheart.00722.2010 21131483 PMC3044062

[B47] Levitan I. B. (1994). Modulation of ion channels by protein phosphorylation and dephosphorylation. Annu. Rev. Physiol. 56, 193–212. 10.1146/annurev.ph.56.030194.001205 7516643

[B48] Li J. Al-Khalili O. Ramosevac S. Eaton D. C. Denson D. D. (2010). Protein-protein interaction between cPLA2 and splice variants of α-subunit of BK channels. Am. J. Physiol. Cell Physiol. 298, C251–C262. 10.1152/ajpcell.00221.2009 19940072 PMC3774341

[B49] Li L. Bhatia M. Zhu Y. Z. Zhu Y. C. Ramnath R. D. Wang Z. J.. (2005). Hydrogen sulfide is a novel mediator of lipopolysaccharide-induced inflammation in the mouse. FASEB J. 19, 1196–1198. 10.1096/fj.04-3583fje 15863703

[B50] Liang G. H. Adebiyi A. Leo M. D. McNally E. M. Leffler C. W. Jaggar J. H. (2011). Hydrogen sulfide dilates cerebral arterioles by activating smooth muscle cell plasma membrane KATP channels. Am. J. Physiol. Heart Circ. Physiol. 300, H2088–H2095. 10.1152/ajpheart.01290.2010 21421823 PMC3119097

[B51] Lide D. R. (1998). CRC Handbook of Chemistry and Physics, 79th Edn. Boca Raton, FL: Taylor & Francis.

[B52] Liu W.-Q. Chai C. Li X.-Y. Yuan W.-J. Wang W.-Z. Lu Y. (2011). The cardiovascular effects of central hydrogen sulfide are related to K(ATP) channels activation. Physiol. Res. 60, 729–738. 21812514 10.33549/physiolres.932092

[B53] Liu Y.-H. Lu M. Hu L.-F. Wong P. T.-H. Webb G. D. Bian J.-S. (2012). Hydrogen sulfide in the mammalian cardiovascular system. Antioxid. Redox Signal. 17, 141–185. 10.1089/ars.2011.4005 22304473

[B54] Łowicka E. Bełtowski J. (2007). Hydrogen sulfide (H_2_S) - the third gas of interest for pharmacologists. Pharmacol. Rep. 59, 4–24. 17377202

[B55] Lu R. Alioua A. Kumar Y. Eghbali M. Stefani E. Toro L. (2006). MaxiK channel partners: physiological impact. J. Physiol. (Lond.) 570, 65–72. 10.1113/jphysiol.2005.098913 16239267 PMC1464300

[B56] Maeda Y. Aoki Y. Sekiguchi F. Matsunami M. Takahashi T. Nishikawa H.. (2009). Hyperalgesia induced by spinal and peripheral hydrogen sulfide: evidence for involvement of Cav3.2 T-type calcium channels. Pain 142, 127–132. 10.1016/j.pain.2008.12.021 19167819

[B56a] Mani S. Untereiner A. Wu L. Wang R. (2014). Hydrogen sulfide and the pathogenesis of atherosclerosis. Antioxid. Redox Signal. 20, 805–817. 10.1089/ars.2013.5324 23582095

[B57] Matsunami M. Kirishi S. Okui T. Kawabata A. (2012). Hydrogen sulfide-induced colonic mucosal cytoprotection involves T-type calcium channel-dependent neuronal excitation in rats. J. Physiol. Pharmacol. 63, 61–68. 22460462

[B58] Millero F. J. Plese T. Fernandez M. (1988). The dissociation of hydrogen sulfide in seawater. Limnol. Oceanogr. 33, 269–274 10.4319/lo.1988.33.2.0269

[B59] Mustafa A. K. Gadalla M. M. Snyder S. H. (2009). Signaling by gasotransmitters. Sci Signal 2:re2. 10.1126/scisignal.268re2 19401594 PMC2744355

[B60] Nagai Y. Tsugane M. Oka J.-I. Kimura H. (2004). Hydrogen sulfide induces calcium waves in astrocytes. FASEB J. 18, 557–559. 10.1096/fj.03-1052fje 14734631

[B61] Olson K. R. (2012). A practical look at the chemistry and biology of hydrogen sulfide. Antioxid. Redox Signal. 17, 32–44. 10.1089/ars.2011.4401 22074253 PMC3342559

[B62] Olson K. R. Whitfield N. L. (2010). Hydrogen sulfide and oxygen sensing in the cardiovascular system. Antioxid. Redox Signal. 12, 1219–1234. 10.1089/ars.2009.2921 19803742

[B63] Peng H. Cheng Y. Dai C. King A. L. Predmore B. L. Lefer D. J.. (2011). A fluorescent probe for fast and quantitative detection of hydrogen sulfide in blood. Angew. Chem. Int. Ed. Engl. 50, 9672–9675. 10.1002/anie.201104236 21882324 PMC3529136

[B64] Pinheiro J. Bates D. DebRoy S. Sarkar D. R Core Team (2013). nlme: Linear and Nonlinear Mixed Effects Models. Available online at: http://CRAN.R-project.org/package=nlme

[B65] R Development Core Team (2011). R: A Language and Environment for Statistical Computing. Vienna: R Foundation for Statistical Computing Available online at: http://www.r-project.org

[B66] Reinhart P. H. Chung S. Martin B. L. Brautigan D. L. Levitan I. B. (1991). Modulation of calcium-activated potassium channels from rat brain by protein kinase A and phosphatase 2A. J. Neurosci. 11, 1627–1635. 1646298 10.1523/JNEUROSCI.11-06-01627.1991 PMC6575393

[B67] Reinhart P. H. Levitan I. B. (1995). Kinase and phosphatase activities intimately associated with a reconstituted calcium-dependent potassium channel. J. Neurosci. 15, 4572–4579. 7790924 10.1523/JNEUROSCI.15-06-04572.1995 PMC6577735

[B68] Renga B. (2011). Hydrogen sulfide generation in mammals: the molecular biology of cystathionine-β- synthase (CBS) and cystathionine-γ-lyase (CSE). Inflamm. Allergy Drug Targets 10, 85–91. 10.2174/187152811794776286 21275900

[B69] Salkoff L. Butler A. Ferreira G. Santi C. Wei A. (2006). High-conductance potassium channels of the SLO family. Nat. Rev. Neurosci. 7, 921–931. 10.1038/nrn1992 17115074

[B70] Savage J. C. Gould D. H. (1990). Determination of sulfide in brain tissue and rumen fluid by ion-interaction reversed-phase high-performance liquid chromatography. J. Chromatogr. 526, 540–545. 10.1016/S0378-4347(00)82537-2 2361993

[B71] Schubert R. Nelson M. T. (2001). Protein kinases: tuners of the BKCa channel in smooth muscle. Trends Pharmacol. Sci. 22, 505–512. 10.1016/S0165-6147(00)01775-2 11583807

[B72] Shibuya N. Koike S. Tanaka M. Ishigami-Yuasa M. Kimura Y. Ogasawara Y.. (2013). A novel pathway for the production of hydrogen sulfide from D-cysteine in mammalian cells. Nat Commun 4, 1366. 10.1038/ncomms2371 23340406

[B73] Shibuya N. Mikami Y. Kimura Y. Nagahara N. Kimura H. (2009). Vascular endothelium expresses 3-mercaptopyruvate sulfurtransferase and produces hydrogen sulfide. J. Biochem. 146, 623–626. 10.1093/jb/mvp111 19605461

[B74] Shipston M. J. Armstrong D. L. (1996). Activation of protein kinase C inhibits calcium-activated potassium channels in rat pituitary tumour cells. J. Physiol. (Lond.) 493(Pt 3), 665–672. 8799890 10.1113/jphysiol.1996.sp021413 PMC1159016

[B75] Sitdikova G. F. Weiger T. M. Hermann A. (2010). Hydrogen sulfide increases calcium-activated potassium (BK) channel activity of rat pituitary tumor cells. Pflugers Arch. 459, 389–397. 10.1007/s00424-009-0737-0 19802723

[B76] Skovgaard N. Gouliaev A. Aalling M. Simonsen U. (2011). The role of endogenous H2S in cardiovascular physiology. Curr. Pharm. Biotechnol. 12, 1385–1393. 10.2174/138920111798280956 22309020

[B77] Sokal R. R. Rohlf F. J. (1995). Biometry: the Principles and Practice of Statistics in Biological Research. New York, NY: W. H. Freeman.

[B78] Stojilkovic S. S. Tabak J. Bertram R. (2010). Ion channels and signaling in the pituitary gland. Endocr. Rev. 31, 845–915. 10.1210/er.2010-0005 20650859 PMC3365841

[B79] Sun Y.-G. Cao Y.-X. Wang W.-W. Ma S.-F. Yao T. Zhu Y.-C. (2008). Hydrogen sulphide is an inhibitor of L-type calcium channels and mechanical contraction in rat cardiomyocytes. Cardiovasc. Res. 79, 632–641. 10.1093/cvr/cvn140 18524810

[B80] Szabó C. (2007). Hydrogen sulphide and its therapeutic potential. Nat. Rev. Drug Discov. 6, 917–935. 10.1038/nrd2425 17948022

[B81] Tang G. Wu L. Wang R. (2010). Interaction of hydrogen sulfide with ion channels. Clin. Exp. Pharmacol. Physiol. 37, 753–763. 10.1111/j.1440-1681.2010.05351.x 20636621

[B82] Tang G. Yang G. Jiang B. Ju Y. Wu L. Wang R. (2013). H2S is an endothelium-derived hyperpolarizing factor. Antioxid. Redox Signal. 19, 1634–1646. 10.1089/ars.2012.4805 23418650

[B83] Telezhkin V. Brazier S. P. Cayzac S. H. Wilkinson W. J. Riccardi D. Kemp P. J. (2010). Mechanism of inhibition by hydrogen sulfide of native and recombinant BKCa channels. Respir. Physiol. Neurobiol. 172, 169–178. 10.1016/j.resp.2010.05.016 20576528

[B84] Telezhkin V. Brazier S. P. Cayzac S. Müller C. T. Riccardi D. Kemp P. J. (2009). Hydrogen sulfide inhibits human BK(Ca) channels. Adv. Exp. Med. Biol. 648, 65–72. 10.1007/978-90-481-2259-2_7 19536466

[B85] Tian L. Coghill L. S. McClafferty H. MacDonald S. H.-F. Antoni F. A. Ruth P.. (2004). Distinct stoichiometry of BKCa channel tetramer phosphorylation specifies channel activation and inhibition by cAMP-dependent protein kinase. Proc. Natl. Acad. Sci. U.S.A. 101, 11897–11902. 10.1073/pnas.0402590101 15280542 PMC511071

[B86] Tian L. Duncan R. R. Hammond M. S. Coghill L. S. Wen H. Rusinova R.. (2001). Alternative splicing switches potassium channel sensitivity to protein phosphorylation. J. Biol. Chem. 276, 7717–7720. 10.1074/jbc.C000741200 11244090

[B87] Treistman S. N. Martin G. E. (2009). BK Channels: mediators and models for alcohol tolerance. Trends Neurosci. 32, 629–637. 10.1016/j.tins.2009.08.001 19781792 PMC4115799

[B88] van Welie I. du Lac S. (2011). Bidirectional control of BK channel open probability by CAMKII and PKC in medial vestibular nucleus neurons. J. Neurophysiol. 105, 1651–1659. 10.1152/jn.00058.2011 21307321 PMC3075294

[B90] Venables W. N. Ripley B. D. (2002). Modern Applied Statistics with S. New York, NY: Springer.

[B91] Wang R. (2010). Hydrogen sulfide: the third gasotransmitter in biology and medicine. Antioxid. Redox Signal. 12, 1061–1064. 10.1089/ars.2009.2938 19845469

[B92] Wang R. (2012). Physiological implications of hydrogen sulfide: a whiff exploration that blossomed. Physiol. Rev. 92, 791–896. 10.1152/physrev.00017.2011 22535897

[B93] Wang R. (2014). Gasotransmitters: growing pains and joys. Trends Biochem. Sci. 39, 227–232. 10.1016/j.tibs.2014.03.003 24767680

[B94] Wang Z.-W. (2008). Regulation of synaptic transmission by presynaptic CaMKII and BK channels. Mol. Neurobiol. 38, 153–166. 10.1007/s12035-008-8039-7 18759010 PMC2706205

[B95] Warenycia M. W. Goodwin L. R. Benishin C. G. Reiffenstein R. J. Francom D. M. Taylor J. D.. (1989). Acute hydrogen sulfide poisoning. Demonstration of selective uptake of sulfide by the brainstem by measurement of brain sulfide levels. Biochem. Pharmacol. 38, 973–981. 10.1016/0006-2952(89)90288-8 2930598

[B96] Weiger T. M. Hermann A. Levitan I. B. (2002). Modulation of calcium-activated potassium channels. J. Comp. Physiol. A Neuroethol. Sens. Neural. Behav. Physiol. 188, 79–87. 10.1007/s00359-002-0281-2 11919690

[B97] White R. E. Schonbrunn A. Armstrong D. L. (1991). Somatostatin stimulates Ca(2+)-activated K+ channels through protein dephosphorylation. Nature 351, 570–573. 10.1038/351570a0 1710783

[B98] Whitfield N. L. Kreimier E. L. Verdial F. C. Skovgaard N. Olson K. R. (2008). Reappraisal of H2S/sulfide concentration in vertebrate blood and its potential significance in ischemic preconditioning and vascular signaling. Am. J. Physiol. Regul. Integr. Comp. Physiol. 294, R1930–R1937. 10.1152/ajpregu.00025.2008 18417642

[B99] Widmer H. A. Rowe I. C. M. Shipston M. J. (2003). Conditional protein phosphorylation regulates BK channel activity in rat cerebellar Purkinje neurons. J. Physiol. (Lond.) 552, 379–391. 10.1113/jphysiol.2003.046441 14561822 PMC2343377

[B100] Wintner E. A. Deckwerth T. L. Langston W. Bengtsson A. Leviten D. Hill P.. (2010). A monobromobimane-based assay to measure the pharmacokinetic profile of reactive sulphide species in blood. Br. J. Pharmacol. 160, 941–957. 10.1111/j.1476-5381.2010.00704.x 20590590 PMC2936000

[B101] Wu S.-N. Wang Y.-J. Lin M.-W. (2007). Potent stimulation of large-conductance Ca2+-activated K+ channels by rottlerin, an inhibitor of protein kinase C-delta, in pituitary tumor (GH3) cells and in cortical neuronal (HCN-1A) cells. J. Cell. Physiol. 210, 655–666. 10.1002/jcp.20866 17133362

[B102] Wu Y. Yang Y. Ye S. Jiang Y. (2010). Structure of the gating ring from the human large-conductance Ca(2+)-gated K(+) channel. Nature 466, 393–397. 10.1038/nature09252 20574420 PMC2910425

[B103] Xie J. McCobb D. P. (1998). Control of alternative splicing of potassium channels by stress hormones. Science 280, 443–446. 10.1126/science.280.5362.443 9545224

[B104] Zhao W. Wang R. (2002). H(2)S-induced vasorelaxation and underlying cellular and molecular mechanisms. Am. J. Physiol. Heart Circ. Physiol. 283, H474–H480. 10.1152/ajpheart.00013.2002 12124191

[B105] Zhao Y. Wei H. Kong G. Shim W. Zhang G. (2013). Hydrogen sulfide augments the proliferation and survival of human induced pluripotent stem cell-derived mesenchymal stromal cells through inhibition of BKCa. Cytotherapy 15, 1395–1405. 10.1016/j.jcyt.2013.06.004 23992829

[B106] Zhou X. B. Arntz C. Kamm S. Motejlek K. Sausbier U. Wang G. X.. (2001). A molecular switch for specific stimulation of the BKCa channel by cGMP and cAMP kinase. J. Biol. Chem. 276, 43239–43245. 10.1074/jbc.M104202200 11514553

[B107] Zhou X.-B. Wulfsen I. Utku E. Sausbier U. Sausbier M. Wieland T.. (2010). Dual role of protein kinase C on BK channel regulation. Proc. Natl. Acad. Sci. U.S.A. 107, 8005–8010. 10.1073/pnas.0912029107 20385812 PMC2867903

[B108] Zhou X. Wulfsen I. Korth M. McClafferty H. Lukowski R. Shipston M. J.. (2012). Palmitoylation and membrane association of STREX controls BK channel regulation by protein kinase C. J. Biol. Chem. 287, 32161–32171. 10.1074/jbc.M112.386359 22843729 PMC3442546

[B109] Zuur A. F. Ieno E. N. Walker N. Saveliev A. A. Smith G. M. (2009). Mixed Effects Models and Extensions in Ecology with R. New York, NY: Springer 10.1007/978-0-387-87458-6

