# Supplementary material for: Phosphorylation of BK channels modulates the sensitivity to hydrogen sulfide (H2S)
Source: Front Physiol. 2014 Nov 12;5:431. doi: 10.3389/fphys.2014.00431 (PMC4228848; doi:10.3389/fphys.2014.00431)
Supplement: Supplementary file 1 [file DataSheet1.DOCX]

Supplementary materials: Amplitude and open dwell time histograms

R-codes for statistical analyses of open probability





Fig.1) GH3 cells: amplitude and open dwell time distributions of BK channels from a single patch in the absence of ATP (ATP0) before (control)and after application of 300 µM sodium hydrosulfide (NaHS). Red lines indicate fit of data; black lines original data. µ = mean channel amplitude in pA, dwell time indicates mean channel open dwell time, Ƭ1 and Ƭ2 are the time constants obtained from the fit to an standard exponential as implemented in Clampfit software with two terms.





Fig.2) GH3 cells: amplitude and open dwell time distributions of BK channels from a single patch in the presence of 1 mM ATP and protein kinase G (PKG) before (control)and after application of 300 µM NaHS. Red lines indicate fit of data; black lines original data. µ = mean channel amplitude in pA, dwell time indicates mean channel open dwell time, Ƭ1 and Ƭ2 are the time constants obtained from the fit to an standard exponential as implemented in Clampfit software with two terms.





Fig.3) GH4 cells: amplitude and open dwell time distributions of BK channels from a single patch in the presence of 1 mM ATP (ATP1) before (control)and after application of 300µM NaHS. Red lines indicate fit of data; black lines original data. µ = mean channel amplitude in pA, dwell time indicates mean channel open dwell time, Ƭ1 and Ƭ2 are the time constants obtained from the fit to an standard exponential as implemented in Clampfit software with two terms.





Fig.4 GH4 cells: amplitude and open dwell time distributions of BK channels from a single patch in the presence of 1 mM ATP (ATP1) and protein kinase A (PKA) before (control)and after application of 300 µM NaHS. Red lines indicate fit of data; black lines original data. µ = mean channel amplitude in pA, dwell time indicates mean channel open dwell time, Ƭ1 and Ƭ2 are the time constants obtained from the fit to an standard exponential as implemented in Clampfit software with two terms.





Fig.5) GH4 STREX cells: amplitude and open dwell time distributions of BK channels from a single patch in the absence of ATP (ATP0) before (control)and after application of 300µM NaHS. Red lines indicate fit of data; black lines original data. µ = mean channel amplitude in pA, dwell time indicates mean channel open dwell time, Ƭ1 and Ƭ2 are the time constants obtained from the fit to an standard exponential as implemented in Clampfit software with two terms.





Fig.6) GH4 STREX cells: amplitude and open dwell time distributions of BK channels from a single patch in the presence of 1 mM ATP (ATP1) before (control)and after application of 300 µM NaHS. Red lines indicate fit of data; black lines original data. µ = mean channel amplitude in pA, dwell time indicates mean channel open dwell time, Ƭ1 and Ƭ2 are the time constants obtained from the fit to an standard exponential as implemented in Clampfit software with two terms.

**R-code example for analyzing open probabilities**

##### Explanation of Data Structure ######

## Demonstration of of data (no 'real' data included!)

## 'data.frame': 'rec'

## original data was acquired/processed/measured with pClamp10/Clampfit10

##

## 7 variables:

## 'Filename': record-ID

## 'Sulfid': control, NaSH

## 'P.open': open probabilities

## 'Dwell.t': dwell times

## 'Ampl': amplitudes

## 'Condition': ATP0, ATP1, PKA, PKC.su, PKC.in, PKG, Stauro, Ocadaic

## 'Celltype': GH3.bk, GH4.bk, GH.strex

###

# Filename Sulfid P.open Dwell.t Ampl Condition Celltype

# 075240n10 control 0.02257 10.600 0.820 ATP0 GH3.bk

# 075240n10 NaHS 0.04176 10.980 0.960 ATP0 GH3.bk

# 075240n11 control 0.01478 11.300 0.940 ATP1 GH3.bk

# 075240n11 NaHS 0.02408 11.890 1.200 ATP1 GH3.bk

# 075240n03 control 0.03240 11.100 0.900 PKA GH3.bk

# 075240n03 NaHS 0.04831 11.160 1.030 PKA GH3.bk

# 07n09002 control 0.00560 11.000 0.760 ATP0 GH4.bk

# 07n09002 NaHS 0.00852 11.260 0.840 ATP0 GH4.bk

# ...................................................................

# 07n09004 control 0.01375 10.000 0.830 Ocadaic GH4.strex

# 07n09004 NaHS 0.00582 9.600 0.800 Ocadaic GH4.strex

###

##### 1. Linear Mixed Model Estimation #####

#### Part 1: set up an linear mixed model and compare several models,

#### in order to test which factors have important influences and

#### to get parameter estimates

##

## for mixed-models with 'random-effects' 'nlme' is usually intalled by default

library(nlme)

## test with a (linear) mixed model if there are significant effects of

## treatment ('condition'), addition of sulfide and/or cell type

## Since there are repeated measurements ('control', 'NaHS')

## a mixed-model should be used.

## Since 0 < P.open < 1, P.open needs to be 'logit'- transformed to normalize it.

#

## define the logit-function

logit <- function(p) log(p / (1 - p) )

## insert transformed variable 'lpo'

rec$lpo <- logit(rec$P.open)

# Additionally 'Dwell time' needs to be normalized with 'log()':

rec$ldt <- log(rec$Dwell.t)

## Just one example for open probabilities:

## Test if there is a significant interaction between Condition and Sulfid

## and let Celltype be an additional factor, test also a "full factorial model"

## Since there are 2 measurements on every individual patch

## (control: w/o NaHS / sulfid: added NaHS ),

## 'Filename' is used as 'random factor'

## 'lme()' 'linear mixed effects' from package "nlme"

## Set up models and compare them:

# Test if a full-factorial model would provide a significant better fit:

# For model comparisons use method 'ML' (maximum likelihood)

# instead of the default estimation method 'REML':

po.mod1 <- lme(lpo ~ Condition * Sulfid + Celltype, random = ~ 1 | Filename,

data = rec, method ="ML")

po.mod2 <- lme(lpo ~ Condition * Sulfid * Celltype, random = ~ 1 | Filename,

data = rec, method ="ML")

# Compare them

anova.lme(po.mod1, po.mod2)

## the second model has a higher log-likelihood, now inspect its terms:

anova(po.mod2)

summary(po.mod2)

##### 2. Multiple Comparisons /Testing specifically for sulfid effects ######

## Part 2: Test sulfide effects in each 'condition', especially adjusted for

## multiple comparisons to avoid alpha-level inflation

##

## Part 1 showed that there is a significant interaction between "condition" and ## "sulfid", meaning that the amount of sulfide effect differs

## according to phosphorylation and presence of ATP, and additionally,

## the three different cell lines differ in their effects ("full-factorial

## model')

## thus the data set is split and analysed for every celltype separately

## Needs Package 'multcomp' (Hothorn et al. 2008)

## if not installed:

# install.packages("multcomp") # should do the job

library(multcomp)

# set-up combined factor-levels for 'sulfid' & 'cond'

rec$sulf.cond <- with(rec, interaction(Sulfid, Condition))

## Create subset for GH3.bk

gh3 <- subset(rec, subset=Celltype=="GH3.bk")

## Set up a lme-model to estimate level means and standard errors

gh3.mod <- lme(lpo ~ sulf.cond, random= ~ 1|Filename, data=gh3)

## Multiple tests with 'glht()' from package "multcomp"

## Define test hypotheses (here just one of several possible ways shown):

hypoth <- c("control.ATP0 - NaHS.ATP0 = 0",

"control.ATP1 - NaHS.ATP1 = 0",

"control.PKA - NaHS.PKA = 0",

"control.PKC.su - NaHS.PKC.su = 0",

"control.PKC.in - NaHS.PKC.in = 0",

"control.PKG - NaHS.PKG = 0",

"control.Stauro - NaHS.Stauro = 0",

"control.Ocadaic - NaHS.Ocadaic = 0")

mult.gh3 <- glht(gh3.mod, linfct=mcp(sulf.cond = hypoth) )

## Get results:

summary(mult.gh3)

## End of File ##
